# Distributed activation energy kinetic modeling of combustion of bagasse char, rice straw char and rice husk char blends

**DOI:** 10.1038/s41598-025-24976-8

**Published:** 2025-11-26

**Authors:** Pritam Kumar, Piyush Chaunsali, Ravikrishnan Vinu

**Affiliations:** 1 Chemical Engineering Department, Indian Institute of Technology Madras, Chennai, 600036 India; 2 Civil Engineering Department, Indian Institute of Technology Madras, Chennai, 600036 India

**Keywords:** Rice husk, Rice straw, Sugarcane bagasse, Biochar blends, Distributed activation energy model, Combustion, Energy science and technology, Engineering, Environmental sciences

## Abstract

Utilizing lignocellulosic biomass agro-residues in the bioenergy sector is an effective way to manage the rising energy needs and minimize the carbon footprint, while significantly reducing the solid waste. This study provides a pathway for the use of lignocellulosic biomass, the biochars derived from them, and their blends for bioenergy generation. This research work investigates the combustion characteristics and kinetics of biochars derived from sugarcane bagasse (BG), rice straw (RS) and rice husk (RH), and binary blends of biochars. The combustion performance was assessed using key parameters such as ignition temperature, peak temperature, burnout temperature and maximum combustion rate. The combustion experiments were conducted in a thermogravimetric analyzer, and the kinetics was studied using distributed activation energy model. Three pseudocomponents, viz., cellulose, hemicellulose and lignin, were considered for biomass, while two pseudocomponents, viz., partially decomposed lignin and carbonaceous matter (char), were considered to model the combustion behaviour of biochars. The results signify that the combustion behaviour of biochars is similar to that of coal. During the combustion of individual biochars and their mixtures, the char decomposition exhibited the highest activation energy (247.6 kJ mol^−1^), while combustion of partially decomposed lignin had the lowest activation energy. Notably, the activation energy for combustion of partially decomposed lignin differed significantly among the biochars and their blends. This was maximum for biochar derived from BG (175.5 kJ mol^−1^) and minimum for biochar derived from RS (130.8 kJ mol^−1^). Importantly, the activation energy of combustion of biochar blends was described by using a binary mixture rule, without fitting any kinetic parameters. Burning time, heat release rate, fuel consumption and ash generation analysis demonstrate that biochars and their blends can be utilized in the existing thermal utilities, and blending biochars derived from different biomass feedstocks is shown to enhance the energy potential of the mixtures.

**Supplementary Information:**

The online version contains supplementary material available at 10.1038/s41598-025-24976-8.

## Introduction

 Biomass is considered as an alternate energy source for the energy-intensive industries owing to its low net carbon emissions. However, raw biomass feedstocks have numerous demerits, such as high moisture content, hydrophilic nature, low bulk density, and low calorific value, restricting their usage at large scales^1,2^. These intrinsic challenges greatly affect logistics and overall energy efficiency. Higher amounts of biomass are needed due to its lower energy density, leading to several problems such as transportation, storage and feed handling in thermochemical conversion, biochemical conversion and co-generation facilities^3,4^. Another prime concern is the higher inherent moisture in biomass, which degrades the processing performance and increases the cost of fuel production. The direct use of biomass as a fuel can be technically difficult due to the presence of higher volatile matter, which tends to increase the flue gas volume, choking of furnaces and exhaust lines due to tar deposition.

Earlier research has shown that biomass must be treated before its utilization for bioenergy production^5,6^. Biomass pyrolysis can be a promising technology for generating clean bioenergy due to enhanced calorific value of biochar, high volatile fraction, and minimal N- and S-associated emissions^7,8^. During low-temperature pyrolysis, the biochemical constituents in biomass such as cellulose, hemicellulose and lignin decompose in a wide range of 250–400 °C resulting in the release of volatiles containing oxygenates and hydrocarbons. Consequently, the volatile-rich biomass transforms into a densified solid, characterized by an increased carbon content and low oxygen and hydrogen content. Energy density of this pre-treated biomass is similar to coal, rendering it more suitable for combustion applications. The condensation of volatile-rich hydrocarbons or tar can yield a chemical feedstock for various applications^5^. In addition, seasonal availability and shortage in supply of biomass of consistent quality are significant obstacles to utilizing biomass as an alternative energy source in industries.

From this perspective, the use of biochar derived from mild pyrolysis of lignocellulosic biomass can overcome the shortcomings of raw biomass and its availability. Agricultural residues such as sugarcane bagasse, rice straw and rice husk are abundantly available in India. The estimated surplus crop residues (in million tons/year) in India like sugarcane bagasse, rice straw and rice husk are 45.78, 44.65 and 6.87, respectively, which corresponds to power equivalent of 4,879 MW_e_, 7,746 MW_e_ and 1,028 MW_e_^9^. Thus, the conversion of these crop residues into biochars, and combustion of blends of biochars derived from diverse biomass feedstocks can offer a solution for using biomass in energy intensive industries .

Numerous studies have reported the co-combustion of coal with different kinds of biomass feedstocks^8–11^. By analyzing the behaviour of biomass-coal combustion at different heating rates using thermogravimetric analysis (TGA), Vhathvarothai et al.^10^ and Issac et al.^12^ found no clear synergistic interaction between biomass and coal during combustion. Magalhães et al.^11^ observed that biomass has superior ignition and combustion reactivity compared to coals. Vamvuka et al.^13^ reported that the interactions between biomass and coal during combustion are less favored, and they ignite and burn separately. Some literature suggests that earlier and rapid combustion issues with biomass can be mitigated by converting them to biochar through pyrolysis^14,15^. After studying the combustion characteristics of biochars obtained at different pyrolysis temperatures (300–500 °C), Chen et al.^15^ observed a significant enhancement in the combustion properties of biochars, particularly at elevated temperatures. Khater et al.^14^ investigated the combustion reactivity of biochar and characterized the microstructure at different pyrolysis temperatures. They found that the biomass surface gradually deteriorates as the functional groups decompose and the microcrystalline structure becomes graphitized at elevated temperatures. This makes the diffusion of O_2_ difficult to break the chemical bonds and release energy. Some researchers have also analyzed the combustion behaviour of biochar and coal, suggesting that the interactions between biochar and coal are more stable and less intense compared to co-firing coal with raw biomass^16–18^. Based on the works carried out by the previous researchers, it is found that the co-combustion of biochar and coal is technically feasible due to the uniform burning profiles of coal and biochar. However, CO_2_ and particulate emissions are major concerns when coal is co-combusted with biochar.

Blending of biochars obtained from different biomass feedstocks can meet the thermal demand of the industrial utilities while also meeting carbon neutrality. Biochar-based combustion in furnaces depends on biochar’s physicochemical characteristics, thermal characteristics, inorganic composition and functional groups in it^19^. Due to the deviation of these properties from the design parameters, which are based on coal’s characteristics, thermal utilities face challenges in utilizing biochar efficiently^19^. Hence, for efficient biochar consumption in thermal utilities, it is necessary to obtain deeper insights on the influence of these parameters on biochar combustion. In thermal utilities, the solid fuel’s heat release rate and burning time, which in turn is impacted by combustion rate, combustible functional groups, volatile matter and fixed carbon of the fuel, are critical parameters. Fuel consumption and ash generation rates vary with the ash content and higher heating value (HHV) of the fuel, respectively. The ash deposition in various zones of the boiler is substantially impacted by inorganic composition of the fuel^20^. Economizer, reheater and superheater tubes of boiler face uneven heat distribution due to larger deviation in combustion temperature of the fuel^21^. Therefore, for efficient utilization of biochar, either as a blend with coal or as an individual fuel in boilers, a thorough assessment of its combustion characteristics needs to be performed, which forms the basis of this study.

Understanding the reactivity and kinetics of fuel combustion is key to the design, modification and optimization of industrial furnaces and combustors^22^. Detailed studies on analysis of combustion behavior of individual biochar and biochar blends are either very limited or unavailable. Therefore, it is important to prioritize the investigation of the combustion behavior of various biochars and their blends. Only few studies have analyzed the combustion reactivity and kinetics of biochar^22–26^. However, these investigations lack the kinetic data typically required for industrial utilities. Distributed activation energy model (DAEM) provides detailed insights on the kinetics of decomposition of solid fuels based on the degradation of the individual chemical constituents. Other integral isoconversional models such as Coats-Redfern, Flynn-Wall-Ozawa and Kissinger-Akharia-Sunose methods can predict biochar combustion only to a certain extent, because they only consider the overall mass loss of the fuel but not the decomposition of its constitutive components^23–25^. Empirical models may offer faster computations but lack the ability to provide mechanistic insights or adaptable performance across diverse feedstocks, especially blends and mixtures. However, DAEM employs a continuous distribution of activation energies associated with the pseudocomponents in order to account for the overlapping multi-stage reactions. This flexibility allows DAEM to model simultaneous processes without oversimplification. In addition, DAEM avoids the common issue of unrealistic activation energy values often observed in global kinetic models due to oversimplification with fewer parameters. DAEM has been successfully validated for the combustion kinetics of few feedstocks including sawdust biochar, wheat straw, rice husk, torrefied forest residue and lignite coal^21,26–28^. These studies show that the model consistently predicts differential TG (DTG) and mass loss behaviour with reasonable accuracy, even at different heating rates. Detailed kinetic analysis of combustion of biochars and their blends is imperative because biochar combustion involves several intricate processes and can only be reasonably understood by using the DAEM method.

Yang et al.^26^ performed the combustion kinetic study of sawdust biochar based on genetic algorithm using double distributed activation energy model. Ma et al.^27^ carried out the kinetic analysis of combustion of rice husk and wheat straw using DAEM method by considering four pseudo components, namely hemicellulose, cellulose, partial lignin and coke, for capturing the experimental mass loss and DTG curves. Bach et al.^22^ performed combustion kinetic modeling of wet-torrefied forest residues using four parallel reaction DAEM to capture the devolatilization of hemicellulose, cellulose and lignin, and char combustion. To the best of the authors’ knowledge, this is the first study to systematically report the combustion kinetics and performance parameters of different lignocellulosic biochars and their blends. In addition to kinetics, an assessment of boiler performance parameters (characteristic combustion temperatures, surface deposition characteristics, burning time, heat release, fuel consumption and ash generation) is also necessary. The biochar combustion kinetic parameters derived using DAEM is expected to be robust as it corresponds to composition-based pseudocomponents. Furthermore, a correlation of basic characteristics of biochars and their blends with the boiler performance parameters provides valuable insights on the use of different biochars in existing energy assets.

The objectives of this work are to understand the combustion kinetics of three lignocellulose-derived biochars, viz., sugarcane bagasse char, rice straw char, rice husk char, and their binary blends. The combustion of raw biomasses, biochars and biochar blends is investigated using TGA, and DAEM is employed for the estimation of kinetic parameters of decomposition of multiple pseudo-components. The obtained combustion kinetic parameters for the biochars and their blends are compared with raw biomasses and coal. Furthermore, various technical feasibility parameters, such as burning time, heat release rate, fuel consumption rate and ash generation, are estimated for the biochars and their mixtures, and compared with those of coal and biomasses. The findings of this work will aid in process development of combustion of biochar blends for use in thermal utilities of energy intensive industries.

## Materials and methods

### Feedstocks collection and Preparation

Sub-bituminous coal was procured from Kajora area, West Bengal, Eastern Coalfield Limited (23.632407°N 87.171593°E), India, and it was crushed to powder form (−500 + 210 μm size). Sugarcane bagasse residue (BG) was collected from the juice processing center at IIT Madras (12.99151ºN 80.23362ºE). BG was subjected to air drying for 3 days to remove the extraneous moisture. Rice straw (RS) and rice husk (RH) were collected from Punjab, India. Biomasses were crushed and ground to fine powders (−500 + 210 μm size) using a job cutter and herbal grinder. All samples were kept in airtight plastic bags to prevent moisture absorption until characterizations and experiments were performed.

### Preparation of Biochar

**Fig. 1:**
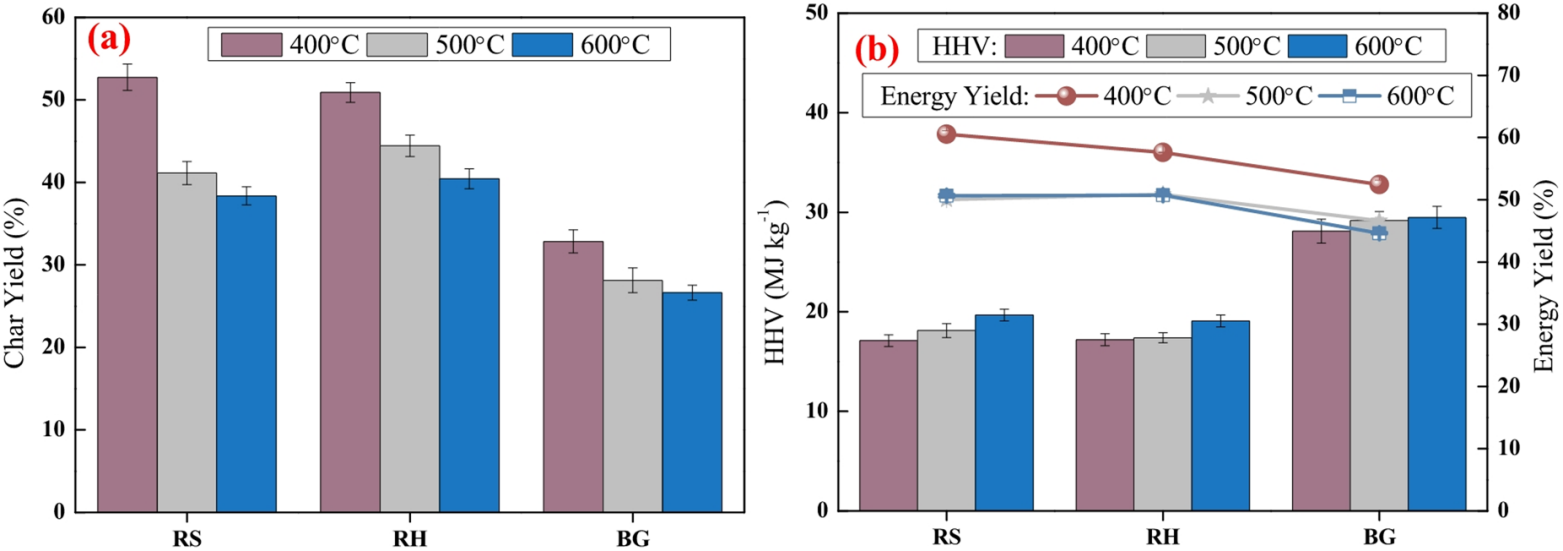
Variations in **(a)** mass yield and **(b)** HHV/energy yield of biochars with pyrolysis temperatures. Energy yield is defined as the product of mass yield and ratio of HHV of biochar to that of biomass.

Biochars from all three biomasses, BG, RS and RH, were made using a tubular pyrolysis unit, depicted in Figure S1. The reactor was made of a quartz tube 800 mm length and 85 mm internal diameter. The temperature was monitored by a K-type thermocouple (± 5 °C). A corundum boat containing 10 g of biomass sample was placed inside the reactor under inert N_2_ environment (100 mL min^−1^). To evacuate the atmospheric air from the reactor, N_2_ was purged for 15 min prior to the commencement of the experiment. The reactor was heated from room temperature (~ 30 °C) to the set temperature (400 °C, 500 °C, 600 °C) at a heating rate of 5 °C min^−1^, and was maintained at the final temperature for 30 min. Finally, it was cooled down to room temperature within 3 h. After the experiment, the residual biochar with the boat was taken out of the reactor and weighed using an analytical balance to determine the biochar yield. Each test was performed thrice to ensure repeatability. The yield, HHV and energy yield of biochars obtained at different pyrolysis temperatures are presented in Fig. 1. Increase in temperature decreases the mass yield of biochar owing to higher extent of devolatilization, while slightly improving the HHV due to the onset of carbonization reactions. However, energy yield is a metric that combines the mass yield and biochar HHV into a single parameter. Evidently, the biochars produced at 400 °C were selected for subsequent combustion studies owing to the high mass and energy yield of biochars from different feedstocks. All biochars were stored in airtight bags for further characterization and experiments. The biochars derived from BG, RS and RH are henceforth denoted as BGC, RSC and RHC, respectively.

### Characterization of feedstocks and biochars

Proximate analysis of coal, biomasses and biochars was performed using a thermogravimetric (TG) analyzer using 7 ± 2 mg of sample as per the ASTM E1131-08 method. Ultimate analysis of all samples was carried out using a CHNS analyzer (Flash 2000, Thermo Fischer Scientific, U.S.A.) following the ASTM D5291 method. The HHV was determined using a bomb calorimeter (IP3, ARICO, India) using 0.5 ± 0.1 g of sample. The salient functional groups present in different samples werev identified using a Fourier transform infrared (FTIR) spectrometer (Agilent Cary 630). The FTIR spectra were obtained in attenuated total reflectance (ATR) mode at a resolution of 4 cm^−1^. Biochemical analysis was performed following the National Renewable Energy Laboratory Analytical Procedure (NREL/TP-510–42618) to determine the hemicellulose, cellulose and lignin content in the biomass feedstocks.

### Preparation of Biochar blends

Different proportions of biochar binary blends, BGC: RSC and BGC: RHC, were prepared using a mortar and pestle. Before mixing , each sample was carefully weighed using an analytical microbalance. Repeated experiments confirmed that the mixtures were representative due to thorough and proper mixing. The biochar binary mixtures were prepared by varying the mass fractions of BGC with RSC and RHC. For example, the nomenclature followed for 20 wt% BGC and 80 wt% RSC is BGC20RSC80. Similarly, BGC40RSC60, BGC60RSC40, BGC80RSC20, BGC20RHC80, BGC40RHC60, BGC60RHC40 and BGC80RHC20 were prepared.

### Combustion experiments using TGA

Combustion experiments for coal, biomasses, biochars and biochar blends were performed in the TG analyzer (SDT Q600, TA instruments). About 7 ± 2 mg of the sample was heated from 30 to 700 °C in presence of zero-air at a flow rate of 100 ml min^−1^, and at sample heating rates of 10 and 20 °C min^−1^.

**Fig. 2:**
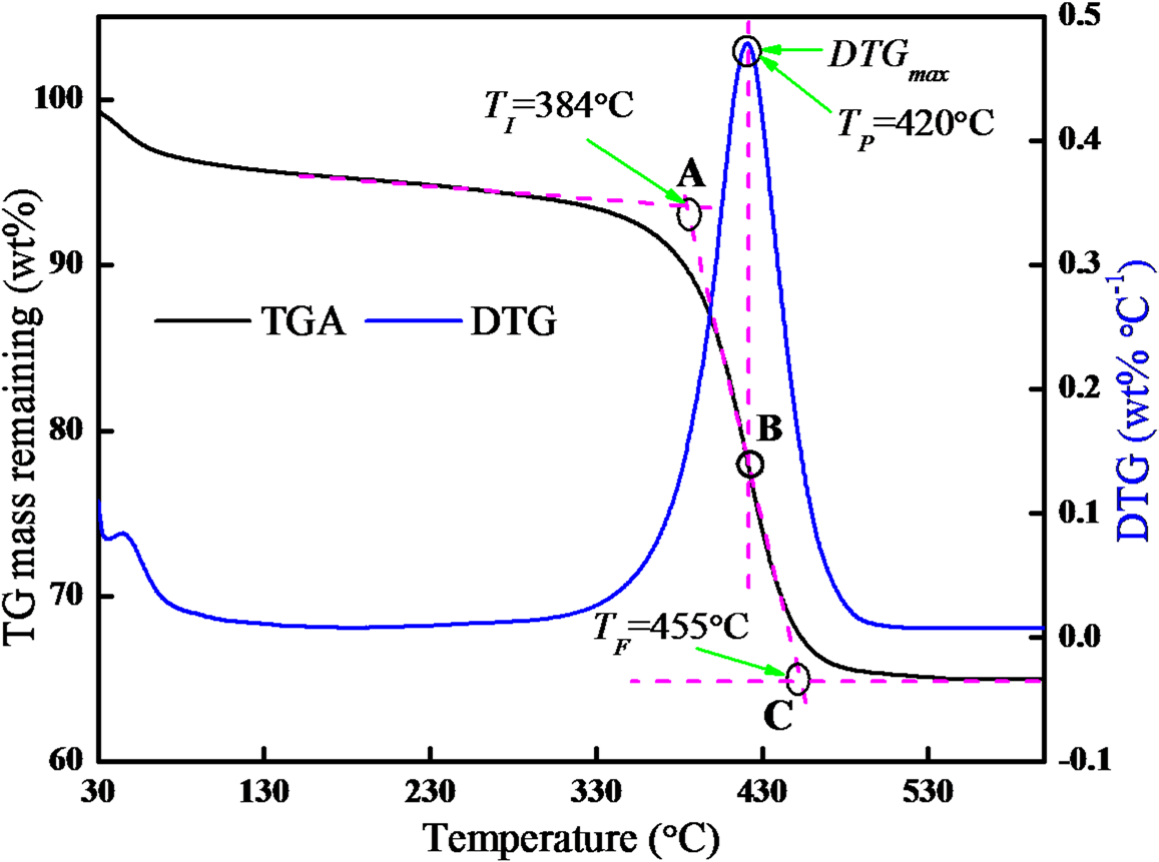
Methodology of extracting the salient combustion characteristics from TGA data. (The data corresponds to combustion characteristics of RSC at 10 °C min^−1^).

The combustion characteristics of all samples were estimated using the TG mass loss and differential TG (DTG) curves, as per well-established procedure^29^. As shown in Fig. 2, a vertical line was drawn from the *DTG*_*max*_ to intersect the TGA curve at point B, and the corresponding temperature at point B is termed peak temperature (*T*_*P*_). Next, a tangent from point B was drawn to intersect the tangent of the initial level of the TGA curve at point A, and the associated temperature at point A is termed ignition temperature (*T*_*I*_). Finally, a tangent drawn from the final level of the TGA curve was made to intersect the tangent drawn from point B at point C, and the corresponding temperature of point C is defined as burnout temperature (*T*_*F*_). The obtained TG-DTG data were used to estimate the combustion kinetics using the DAEM method.

### Combustion kinetics using distributed activation energy model (DAEM)

To elucidate the intricate decomposition mechanism of solid fuels, a multiple parallel DAEM is employed to fit the TG-DTG profiles. This approach is highly reliable due to its robustness in estimating kinetic parameters from diverse thermochemical reactions^30^. The degree of conversion (*α*) is determined based on the TG data using Eq. (1)^31^.

1 $$\:\alpha\:=\:\frac{({m}_{i0}-{m}_{it})}{\left({m}_{i0}-{m}_{f}\right)}$$

where, $\:{m}_{i0}$ is the initial sample mass, $\:{m}_{it}$ is the instantaneous sample mass and $\:{m}_{f}$ is the residual mass at the end of combustion.

In DAEM, the sample is considered to consist of multiple pseudo-components, each decomposing over a spectrum of activation energies following a Gaussian distribution. For a sample consisting of ‘q’ pseudo-components, α is determined by DAEM using Eq. (2)^32^.

2 $$\:\alpha\:=1-\:\sum\nolimits_{i=1}^{q}\left\{{p}_{i}{\int\:}_{0}^{\infty\:}exp\left[-{\int\:}_{{T}_{0}}^{T}\frac{f}{\beta\:}exp\left(\frac{{E}_{a,i}}{R\times\:T}\right)dT\right]f\left({E}_{a,i}\right)d{(E}_{a,i})\right\}$$

In the above expression, $\:{p}_{i}$ is the mass fraction of individual pseudocomponent ‘i’ in the sample, $\:{E}_{a}$ is the activation energy (kJ mol^−1^), *f* is the frequency factor (min^−1^), *β* is the heating rate (K min^−1^), T is the temperature (K) and *f(*$\:{E}_{a})$ is the continuous function of $\:{E}_{a}$ modeled using Gaussian distribution.

The model DTG, $\:\frac{d\alpha\:}{dT}$, is derived by differentiating Eq. (2) with respect to T, and is given as Eq. (3).

3 $$\:\frac{d\alpha\:}{dT}=\sum\nolimits_{i=1}^{q}{\int\:}_{0}^{\infty\:}{p}_{i}\frac{f}{\beta\:}exp\left[-\frac{{E}_{a,i}}{R\times\:T}-\:{\int\:}_{{T}_{0}}^{T}\frac{f}{\beta\:}exp\left(-\frac{{E}_{a,i}}{R\times\:T}\right)dT\right]f\left({E}_{a,i}\right)d\left({E}_{a,i}\right)$$

The Gaussian distribution function *f(*$\:{E}_{a,i}$*)* for the i^th^ pseudocomponent of a sample is given by Eq. (4).

4 $$\:f\left({E}_{a,i}\right)=\frac{1}{{{\upsigma\:}}_{i}\sqrt{2\pi\:}}exp\left(\frac{-{\left({E}_{a,i\:}-{E}_{a0}\right)}^{2}}{2{{\sigma\:}_{i}}^{2}}\right)$$

where, $\:{E}_{a0}$ is the mean activation energy (kJ mol^−1^) and $\:{\sigma\:}_{i}$ is the standard deviation of $\:{E}_{a,i}$ for the i^th^ pseudocomponent. The temperature integral of Eqs. (2,3) has no analytical solution, and has to be approximated using an analytical function given by Eq. (5)^33^.

5 $$\:{\int\:}_{0}^{T}exp\left(-\frac{{E}_{a}}{R\times\:T}\right)dT=\:\frac{{R\times\:T}^{2}}{{E}_{a}}exp\left(-\frac{{E}_{a}}{R\times\:T}\right)\frac{\left(0.99954{E}_{a}+0.58058R\times\:T\right)}{({E}_{a}+2.54R\times\:T)}$$

The model DTG, as given in Eq. (3), was estimated using ‘*α*’ data of TGA at 10 °C min^−1^ based on the pattern search objective function of MATLAB (R2023a). Here, the following parameters, $\:{E}_{a,i}$, $\:{f}_{i}$, $\:{\sigma\:}_{i}$ and $\:{p}_{i}$, were optimized for each pseudocomponent of the sample. The objective functions, $\:{OF}_{DTG}$ for *DTG* and $\:{OF}_{\alpha\:}$ for *α*, are given by Eqs. (6,7).

6 $$\:{OF}_{DTG}=\sum\nolimits_{i=1}^{N}{\left[{\left(\frac{d\alpha\:}{dT}\right)}_{i,exp}-{\left(\frac{d\alpha\:}{dT}\right)}_{i,cal}\right]}^{2}$$

7 $$\:{OF}_{\alpha\:}=\sum\nolimits_{i=1}^{N}{\left[{\alpha\:}_{i,exp}-{\alpha\:}_{i,cal}\right]}^{2}$$

In the above equations, $\:N$ is the number of data points used for the estimation of model *α* and DTG using Eqs. (2,3). The fit% for model *DTG* and *α* were estimated based on the average deviation between experimental and calculated curves of *DTG* and *α* using Eqs. (8,9).

8 $$\:{Fit\:\left(\%\right)}_{DTG}=\left(1-\left(\frac{\sqrt{\frac{{OF}_{DTG}}{N}}}{{\left(\frac{d\alpha\:}{dT}\right)}_{max}}\right)\right)\times\:100\%$$

9 $$\:{Fit\:\left(\%\right)}_{\alpha\:}=\left(1-\left(\sqrt{\frac{{OF}_{\alpha\:}}{N}}\right)\right)\times\:100\%$$

In the above expression, $\:{\left(\frac{d\alpha\:}{dT}\right)}_{max}$ is the maximum experimental value of *DTG*. The fit% should be high for optimal set of parameters of the pseudocomponents^34^. In this work, the parameters corresponding to every pseudocomponent ($\:{E}_{a,i}$, $\:{f}_{i}$, $\:{\text{a}\text{n}\text{d}\:\sigma\:}_{i}$) were optimized to minimize the $\:{OF}_{DTG}$ using Eq. (6), and to better predict the *DTG* value from DAEM using Eq. (3). Once the optimized parameters for *DTG* were obtained, they were used as inputs for the estimation of *α* using Eq. (2). The objective function ($\:{OF}_{\alpha\:})$ and fitness parameter $\:\left({Fit\left(\%\right)}_{\alpha\:}\right)$ were estimated using Eqs. (7, 9) to know the fitting quality of model ‘*α*’. After getting a good fit% for ‘*α*’ and ‘*DTG*’ at a heating rate of 10 °C min ^-1^ , the optimized parameters were used to predict the experimental *α* and *DTG* data at 20 °C min^ -1^, thus ensuring the robustness of the model parameters ($\:{E}_{a,i}$, $\:{f}_{i}$, $\:{\text{a}\text{n}\text{d}\:\sigma\:}_{i}$). It is important to note that pseudocomponent concentrations ($\:{p}_{i})$ were not optimized in this study. For individual biomass and biochar, pseudocomponent concentrations were taken based on their biochemical compositions, while weighted average compositions were taken for pseudocomponents of the biochar blends.

## Results and discussion

### Characterization of coal, biomasses and biochars

The physicochemical characteristics of BG, RS and RH,  and the biochars derived from them, BGC, RSC and RHC, respectively, are compared to that of coal in Table 1. BG contains a higher amount of volatile matter (VM) and fixed carbon (FC) along with a lower ash compared to other biomasses, while RS is rich in ash. Biochars contain low VM, high FC and ash compared to biomasses. Wang et al.^35^ observed similar variations in biochar characteristics compared to raw feedstocks. High FC means enhanced energy density of biochars owing to the loss of lighter volatile matter during pyrolysis. BGC contains higher FC (48.5%) compared to RHC and RSC due to the smaller fraction of ash in it. RSC has minimum VM and maximum ash, and its proximate characteristics are quite similar to coal. The ultimate analysis results show that BG has a higher amount of carbon, hydrogen and oxygen compared to RS and RH. Higher carbon and hydrogen promote higher energy density of the feedstock. Higher oxygen content in the feedstock promotes the release of CO and CO_2_ during pyrolysis via decarbonylation and decarboxylation reactions^36^. Biochars are characterized by lower amount of hydrogen and oxygen with elevated carbon levels than that of biomasses. Similar change in carbon, hydrogen and oxygen content is reported by Uzoagba et al.^37^ for biochars prepared at various pyrolysis temperatures. The decline in hydrogen and oxygen in biochars is due to the mild degradation of hemicellulose, cellulose and lignin present in biomass through the release of low molecular weight volatile compounds like C_1_-C_3_ oxygenates, CO, CO_2_ and H_2_O^38^. The ultimate characteristics of BGC in terms of carbon and hydrogen are better than that of coal, whereas the elemental composition of RSC is similar to coal. Biochars possess higher HHV compared to biomasses. HHV of BGC is found to be significantly higher than RHC and RSC owing to higher FC and hydrogen content in it.

From biochemical analysis, BG has higher hemicellulose and cellulose, while RH has higher lignin. In addition, RH contains elevated level of extractives. Hemicellulose produces more gaseous products and residual char than cellulose, whereas cellulose devolatilization products contribute to bio-oil yield^39,40^. Lignin increases the char yield due to aromatization reactions during pyrolysis, and mostly produces aromatic and phenolic chemicals in the bio-oil. Extractives decompose to produce polyphenols, fatty acids and secondary cracking products like methanol at higher temperatures, and promote the formation of H_2_O, CO_2_ and CO at lower temperatures^40^.

**Fig. 3:**
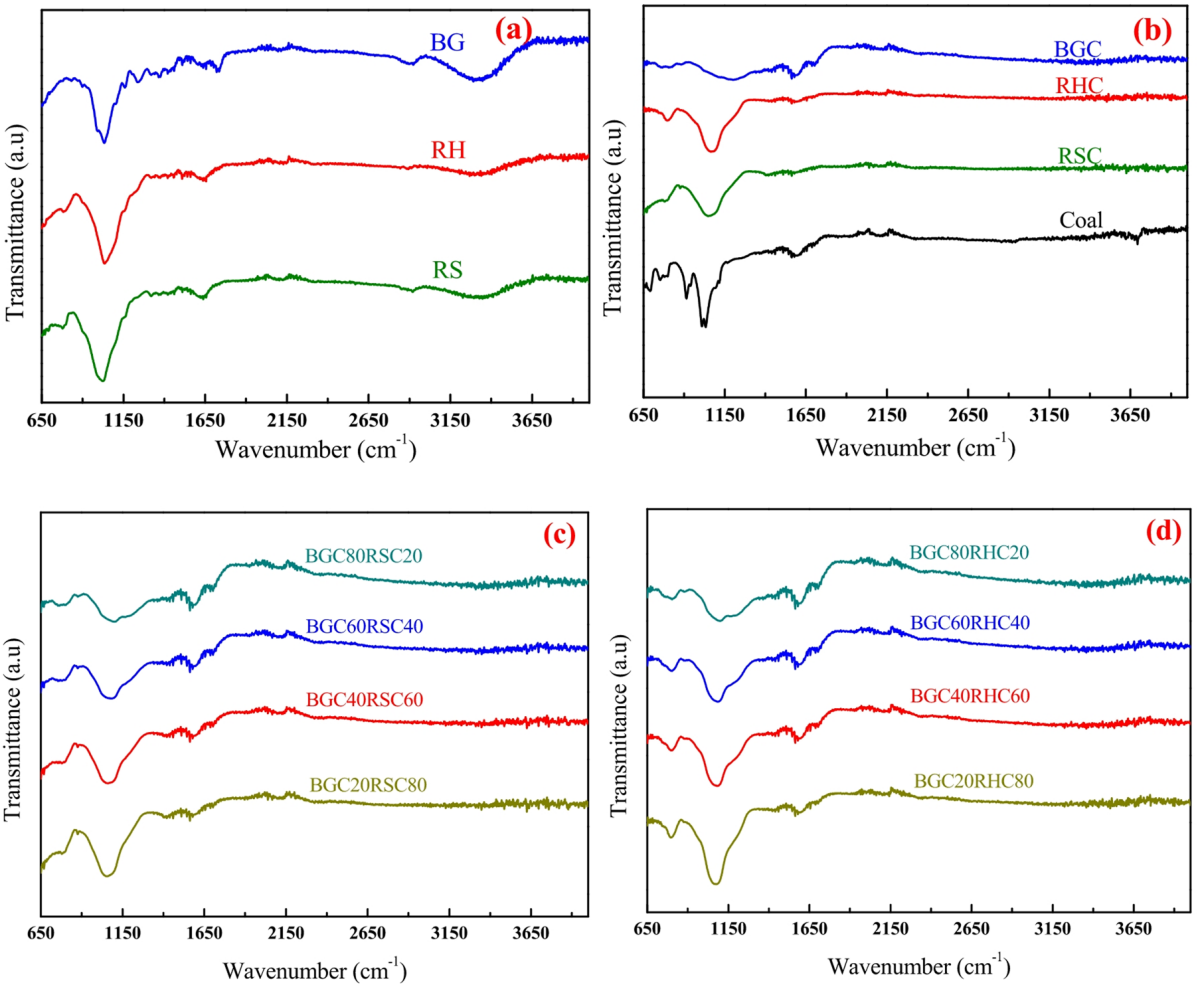
FTIR spectra of **(a)** biomasses, **(b)** biochars and coal, **(c)** BGC-RSC blend and **(d)** BGC-RHC blend.

FTIR spectra of biomasses, biochars, biochars blends and coal are illustrated in Fig. 3(a-b). The following functional group vibrations are evident: O-H stretch (3860 cm^−1^ and 3350 cm^−1^), C-H stretch (2900 cm^−1^), C ≡ C alkyne stretch (2100 cm^−1^), aromatic C = O stretch (1730 cm^−1^ and 1720 cm^−1^), C = O amide stretch (1660 cm^−1^), C = C stretch (1520 cm^−1^ and 1590 cm^−1^), aromatic NO_2_ axisymmetric stretch (1520 cm^−1^ and 1590 cm^−1^), N-O stretch (1370 cm^−1^), aromatic C-H in-plane bend (1250 cm^−1^), aromatic C-H in-plane bend (1100 cm^−1^), C-O stretch (1190 cm^−1^, 1060 cm^−1^ and 1025 cm^−1^), C-N stretch (1190 cm^−1^, 1060 cm^−1^ and 1025 cm^−1^), =C-H out-of-plane bend (911 cm^−1^) and aromatic C-H out-of-plane bend (786 cm^−1^)^41–45^.

**Table 1:** Proximate, ultimate analysis and HHV of biomasses, biochars and coal.

	BG	RS	RH	BGC	RSC	RHC	Coal
Proximate analysis (wt%, db)							
VM	86.0 ± 0.9	61.9 ± 0.7	74.4 ± 0.7	44.2 ± 0.9	23.3 ± 0.8	29.1 ± 0.7	19.8 ± 0.6
FC	11.3 ± 0.5	8.9 ± 0.6	9.6 ± 0.2	48.5 ± 0.7	28.6 ± 0.5	32.5 ± 0.8	33.7 ± 0.9
Ash	2.7 ± 0.1	29.2 ± 0.4	16.0 ± 0.27	7.3 ± 0.2	48.1 ± 0.5	38.4 ± 0.6	46.5 ± 0.5
HHV (MJ kg^−1^)	17.6 ± 1.2	14.9 ± 0.8	15.2 ± 0.9	28.1 ± 1.5	17.1 ± 0.6	17.2 ± 0.6	17.6 ± 1.2
Ultimate analysis (wt%, db)							
C	60.7 ± 1.2	31.6 ± 0.9	43.8 ± 1.0	66.9 ± 1.4	30.7 ± 1.1	43.4 ± 0.9	34.3 ± 1.2
H	6.0 ± 0.2	2.9 ± 0.1	4.3 ± 0.1	4.1 ± 0.2	1.9 ± 0.1	3.2 ± 0.2	1.6 ± 0.1
N	0.3 ± 0.01	0.4 ± 0.1	0.8 ± 0.01	4.0 ± 0.3	3.1 ± 0.2	2.9 ± 0.2	1.1 ± 0.1
O^bd^	30.3	35.9	35.1	17.7	16.2	12.1	16.5
Biochemical analysis (wt%, adb)							
Hemicellulose	25.7 ± 0.9	19.4 ± 1.6	12.4 ± 0.4	-	-	-	-
Cellulose	42.6 ± 2.9	33.3 ± 3.2	32.6 ± 2.4				
Lignin	20.2 ± 1.6	14.5 ± 0.8	23.1 ± 1.2				
Extractives	4.3 ± 0.2	2.6 ± 0.7	10.9 ± 0.5				

db: dry basis, adb: air dried basis, O^bd^ = 100 – (C + H + N + S + Ash).

Several peaks in the FTIR spectra of the biomasses, biochars, biochars blends and coal can be ascribed to the presence of alcohol, phenol, carboxylic acid, methoxy groups of methyne, esters, carbohydrates, aldehydes, ketones, protein and amides. Importantly, some peaks are absent in the biochars (Fig. 3b) because low molecular volatile organic compounds are removed from the biomass during pyrolysis. Additionally, some long-chain hydrocarbons undergo structural transformation due to thermal cracking^19^. Consequently, biomass and biochars have distinct intermolecular bonding. The wider band around 3350 cm^−1^, typically associated with O-H functional groups, is absent in the biochars due to dehydration reactions. The peaks in the spectra of biochars, corresponding to C = O stretch, aromatic C-H in-plane bend, C = C stretch and aromatic C-H out-of-plane bend, signify the presence of hydrocarbon and aromatic compounds. The broader peaks in the spectra of biochars associated with C-O and C-N stretch (1060 cm^−1^ and 1193 cm^−1^) are attributed to the increased fixation of carbon and nitrogen in biochars (Table 1).

Peaks corresponding to C = C stretch and aromatic NO_2_ unsymmetric stretch for BGC are broader due to the presence of higher carbon and nitrogen in BGC than in RSC and RHC. For BGC: RSC and BGC: RHC blends, minor peaks corresponding to C = C stretch and aromatic C–H out-of-plane bend are seen. These minor peaks remain unchanged regardless of the BGC mass fraction, indicating minimal or non-beneficial interactions among the individual biochar samples. C–O stretching vibration originates from the ether linkages and C–O bonds in the lignin structure, while aromatic C–H in-plane bend originates from the guaiacyl and syringyl units^46^. Peaks related to C–O stretching and aromatic C–H in-plane vibrations become more intense and broader as the proportion of BGC increases in both blends, which signifies possible additive effects in functional groups. It is anticipated that higher decomposition energy will be required with the increase of BGC mass fraction in the blends. Overall, the FTIR spectra corresponding to different functional groups are similar for coal, biochars and biochar blends.

**Table 2:** Ash composition (wt%) and deposition indices of coal and biochars.

Sample	SiO_2_	Al_2_O_3_	Fe_2_O_3_	TiO_2_	CaO	Na_2_O	K_2_O	MgO	*BA*_*I*_	*S*_*V*_
Coal	58.9	27.9	2.95	1.86	0.28	0.12	1.73	0.72	0.07	93.7
BGC	28.0	0.48	4.56	0	7.78	0.19	28.63	6.97	1.69	59.2
RSC	58.2	9.13	6.60	0.62	6.05	0.97	12.71	2.31	0.42	79.5
RHC	89.8	0.88	0.44	0	1.19	0	5.42	0.29	0.08	97.9

Ash composition of coal, BGC, RSC and RHC are reported in Table 2. RHC ash contains higher SiO_2_ (89.8 wt%) followed by coal (58.9 wt%), RSC (58.2 wt%) and BGC (28.0 wt%). Al_2_O_3_ is significantly higher (27.9 wt%) in coal compared to RSC (9.13 wt%), RHC (0.88 wt%) and BGC (0.48 wt%). BGC and RSC contain higher amount of alkali oxides like K_2_O, CaO, Fe_2_O_3_ and MgO than that in coal. Variation in mineral composition impacts the combustion characteristics along with slagging and fouling behavior of ash. Based on the ash composition, the base-to-acid index (*BA*_*I*_) and slagging viscosity (*S*_*V*_) were calculated using the following empirical Eqs.^47,48^.

10 $$\:{BA}_{I}=\:\frac{({\text{F}\text{e}}_{2}{\text{O}}_{3}+\:{\text{N}\text{a}}_{2}\text{O}+\:{\text{K}}_{2}\text{O}+\text{C}\text{a}\text{O}+\text{M}\text{g}\text{O})}{{\text{S}\text{i}\text{O}}_{2}+\:{\text{A}\text{l}}_{2}{\text{O}}_{3}+\:{\text{T}\text{i}\text{O}}_{2}}$$

11 $$\:{S}_{V}=\:\frac{{SiO}_{2}}{{\text{S}\text{i}\text{O}}_{2}+\:{\text{F}\text{e}}_{2}{\text{O}}_{3}+\:\text{C}\text{a}\text{O}+\text{M}\text{g}\text{O}}$$

*BA*_*I*_ and *S*_*V*_ of coal and biochars are presented in Table 2. *BA*_*I*_ signifies slagging tendency of fuel during combustion. Specifically, *BA*_*I*_ < 0.50 infers low slagging tendency, 0.5–1.0 infers moderate slagging , and *BA*_*I*_ > 1.0 indicates higher slag formation^48^. *S*_*V*_ value more than 78 signifies low slag formation , *S*_*V*_ value in the range of 66–78 indicates moderate slagging , and *S*_*V*_ value < 66 signifies high slagging tendency^48^. *BA*_*I*_ is highest for BGC (1.69) and lowest for RHC (0.08), *S*_*V*_ is lowest for BGC (59.2) and highest for RHC (97.9). RSC has moderate *BA*_*I*_ and *S*_*V*_. Slagging behaviour of RSC and RHC is similar to coal, while BGC has a higher slagging tendency along with higher calorific value. Overall, it can be anticipated that blending of RSC and RHC with BGC is a possible strategy to minimize the high slagging caused by the use of only bagasse in the boilers. This would also improve the HHV of fuel mixtures, and could be a potential replacement of coal.

### Combustion study using TGA

**Fig. 4:**
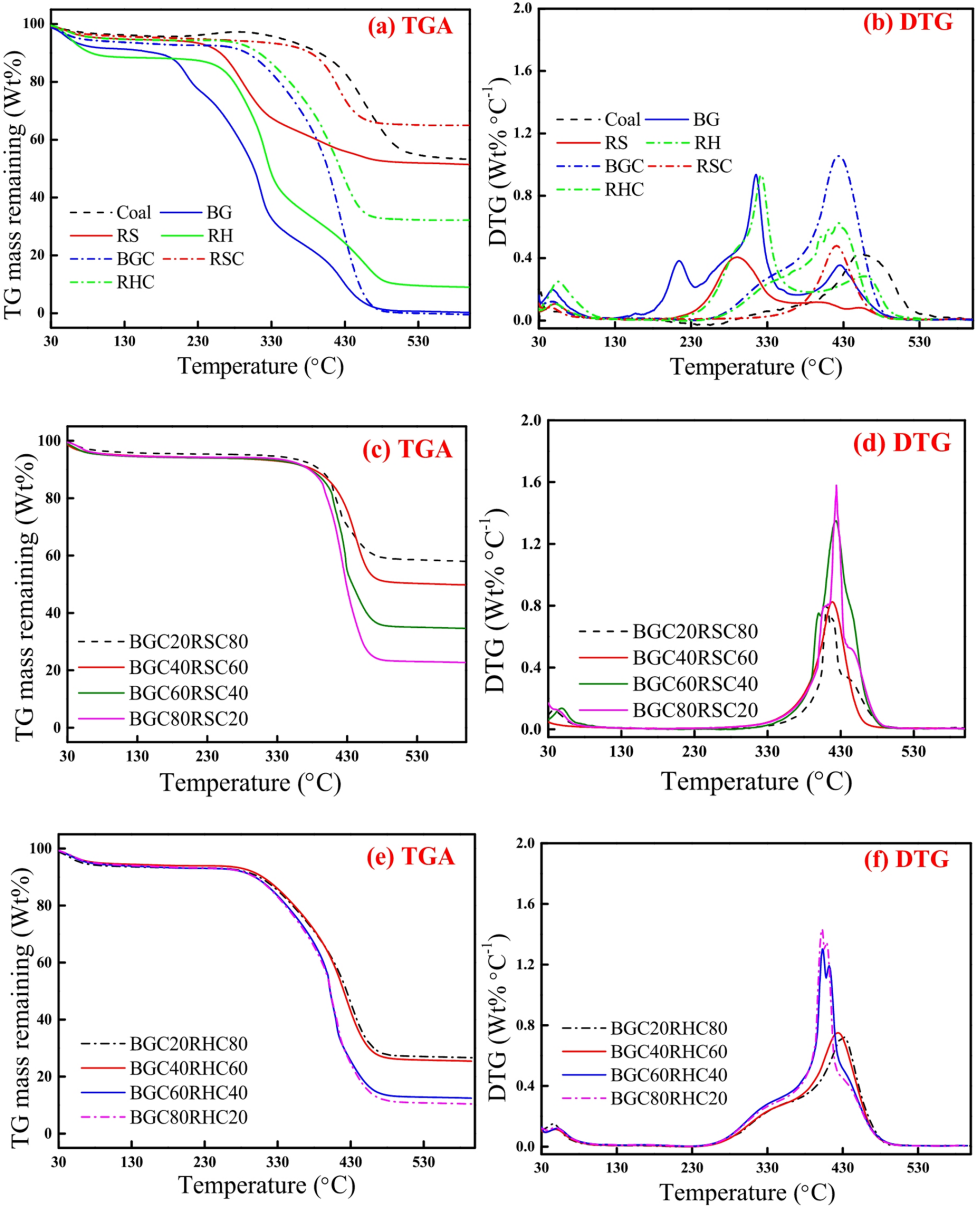
TG-DTG plots for combustion of **(a, b)** coal, BG, RS, RH, BGC, RSC and RHC, **(c, d)** binary blends of BGC: RSC, and **(e, f)** binary blends of BGC: RHC at 10 °C min^−1^.

 Figure 4 depicts the TG mass loss and DTG profiles for the combustion of coal, biomasses, biochars and binary blends of biochar at 10 °C min^−1^. It is evident from Fig. 4 (a-b) that all three biomasses (BG, RS and RH) exhibit distinct combustion characteristics due to differences in hemicellulose, cellulose and lignin content in them. Biochars exhibit distinct combustion profiles than the corresponding biomasses due to the prior removal of hemicellulose and partial devolatilization of cellulose during pyrolysis, which relatively enhances the lignin and FC fraction in them. Individual TG curves signify that with the rise in temperature up to 150 °C, about 5–12 wt% of mass loss is observed, which is attributed to the release of inherent moisture. Rapid mass loss began after 180–220 °C for biomasses and 280–350 °C for biochars, signifying the initiation of the combustion reaction. Similar distinct ignition temperature values for biomass and biochar are observed by Aich et al. ^19^ . Theoretically, this critical point is defined as the ignition temperature (*T*_*I*_), as described in Sect. “Combustion experiments using TGA”. The derivative mass loss profile attains the maximum value at peak temperature (*T*_*P*_), where the combustion rate (*DTG*_*max*_) is maximum. After 450–500 °C, mass loss attains a saturation depending on the composition of the feedstock, inferring the total burnout of the combustible matter and the onset of burnout temperature (*T*_*F*_). Combustion profile of coal is similar to biochars.

The combustion parameters are presented in Table 3. *T*_*I*_ of BG is lowest (188 °C) compared to RS (198 °C) and RH (209 °C). Similarly, *T*_*F*_ of BG is smaller (418 °C) as compared to RS (421 °C) and RH (435 °C). DTG peaks for BG are higher in magnitude and the associated peak temperatures are lower than that of RS and RH. These observations can be attributed to the presence of higher VM, hemicellulose and cellulose and lower ash content in BG. *T*_*I*_, *T*_*P*_ and *T*_*F*_ of biochars are notably higher than the respective biomasses, which can be attributed to the formation of more graphitized microcrystalline structures that are more ordered and carbon-rich, which makes the biochars less reactive compared to raw biomass^14^. These findings signify that biochars exhibit stable combustion behaviour as compared to raw biomass. Thus, biochar usage can mitigate the early and rapid combustion problems associated with the gas phase volatiles which is unavoidable in biomass combustion. The combustion profiles of all biochars exhibit a single DTG peak, analogous to coal combustion, whereas biomass combustion shows multiple DTG peaks. The single DTG peak in biochar combustion results from char oxidation and the combustion of residual lignin^49^. Among biochars, RSC exhibits higher *T*_*I*_ (384 °C), *T*_*F*_ (455 °C) and lower *DTG*_*max*_ (0.48 wt% ºC^−1^) compared to BGC and RHC. This can be attributed, atleast partially, to higher ash and lower VM in RSC. These parameters of RSC are somewhat similar to coal combustion (*T*_*I*_: 387 °C, *T*_*F*_: 476 °C and *DTG*_*max*_: 0.44 wt% ºC^−1^).

**Table 3:** Combustion characteristics for coal, biochars and binary blends of BGC: RSC and BGC: RHC.

Samples	*T*_*I*_ (°C)	*T*_*P*_ (°C)	*T*_*F*_ (°C)	*DTG*_*max*_ (wt% °C^−1^)
Coal	387 ± 0.9	453 ± 1.0	476 ± 1.3	0.44 ± 0.03
BG	188 ± 0.9	213 ± 1.1, 314 ± 0.98,425 ± 1.1	418 ± 1.2	0.39 ± 0.02, 0.93 ± 0.04, 0.36 ± 0.03
RS	198 ± 0.9	290 ± 1.1, 396 ± 1.2, 451 ± 1.0	421 ± 1.1	0.41 ± 0.02, 0.12 ± 0.005, 0.08 ± 0.007
RH	209 ± 1.3	321 ± 1.0, 457 ± 1.2	435 ± 1.2	0.91 ± 0.01, 0.28 ± 0.01
BGC	334 ± 1.2	423 ± 0.95	440 ± 1.3	1.05 ± 0.04
RSC	384 ± 0.9	420 ± 1.1	455 ± 1.2	0.48 ± 0.02
RHC	342 ± 0.9	423 ± 1.2	446 ± 1.1	0.62 ± 0.03
BGC20RSC80	383 ± 0.9	411 ± 1.1, 417 ± 1.0	454 ± 1.2	0.81 ± 0.02, 0.72 ± 0.03
BGC40RSC60	380 ± 1.0	418 ± 1.2	454 ± 1.3	0.82 ± 0.01
BGC60RSC40	380 ± 1.0	423 ± 1.3	454 ± 1.1	1.34 ± 0.05
BGC80RSC20	377 ± 1.0	425 ± 1.1	446 ± 1.2	1.58 ± 0.04
BGC20RHC80	363 ± 1.1	430 ± 1.4	452 ± 1.3	0.72 ± 0.02
BGC40RHC60	361 ± 1.1	424 ± 1.2	452 ± 1.4	0.75 ± 0.02
BGC60RHC40	356 ± 1.2	403 ± 1.2, 413 ± 1.1	452 ± 1.6	1.30 ± 0.03, 1.18 ± 0.01
BGC80RHC20	356 ± 1.0	402 ± 1.0, 409 ± 1.1	452 ± 1.2	1.43 ± 0.04, 1.34 ± 0.02

With the rise of BGC mass fraction in the BGC: RSC blend, the TG curves slightly shifted to the left side, inferring somewhat rapid ignition and burnout (Fig. 4c). However, *DTG*_*max*_ gradually increased, signifying the rapid burning of fuel. Similar observation is also confirmed from Table 3, wherein with the increase of BGC mass fraction from 20 wt% (BGC20RSC80) to 80 wt% (BGC80RSC20), *T*_*I*_ and *T*_*F*_ decreased from 383 °C and  454 °C to 377 °C and 446 °C, respectively. With the increase of BGC mass fraction in BGC: RHC blend, *T*_*I*_ and *T*_*P*_ decreased, while *T*_*F*_ was invariant. The optimal blend ratio for any thermal utility application is determined by assessing how well the fuel blend integrates with the existing system design.

Since combustion characteristics are non-additive, variations in the range of ± 10–20 °C in *T*_*I*_, *T*_*P*_ and *T*_*F*_ is observed as compared to the values for the raw feedstock. However, deviations beyond this range can lead to combustion issues, such as premature or delayed ignition, unburned hydrocarbons in the flue gas, and increased fly ash formation^50^. This shows that any blend ratio of RSC or RHC with BGC can be used in the thermal utility without significant modification, as the critical characteristics are quite closer to that of coal.

To evaluate the presence and effect of synergistic interactions during combustion of BGC-RSC and BGC-RHC blends, a standard deviation (*S.D* %) metric for combustion is defined as per Eqs. (12–13).

12 $$\:S.D\:\left(\%\right)=\:\frac{\:{T}_{exp}-{T}_{cal}}{{T}_{exp}}\times\:100$$

13 $$\:{T}_{cal}=\:\frac{\left({w}_{1}\times\:{T}_{exp,1}\right)+({w}_{2}\times\:{T}_{exp,2})}{({w}_{1}+\:{w}_{2)}}$$

**Fig. 5:**
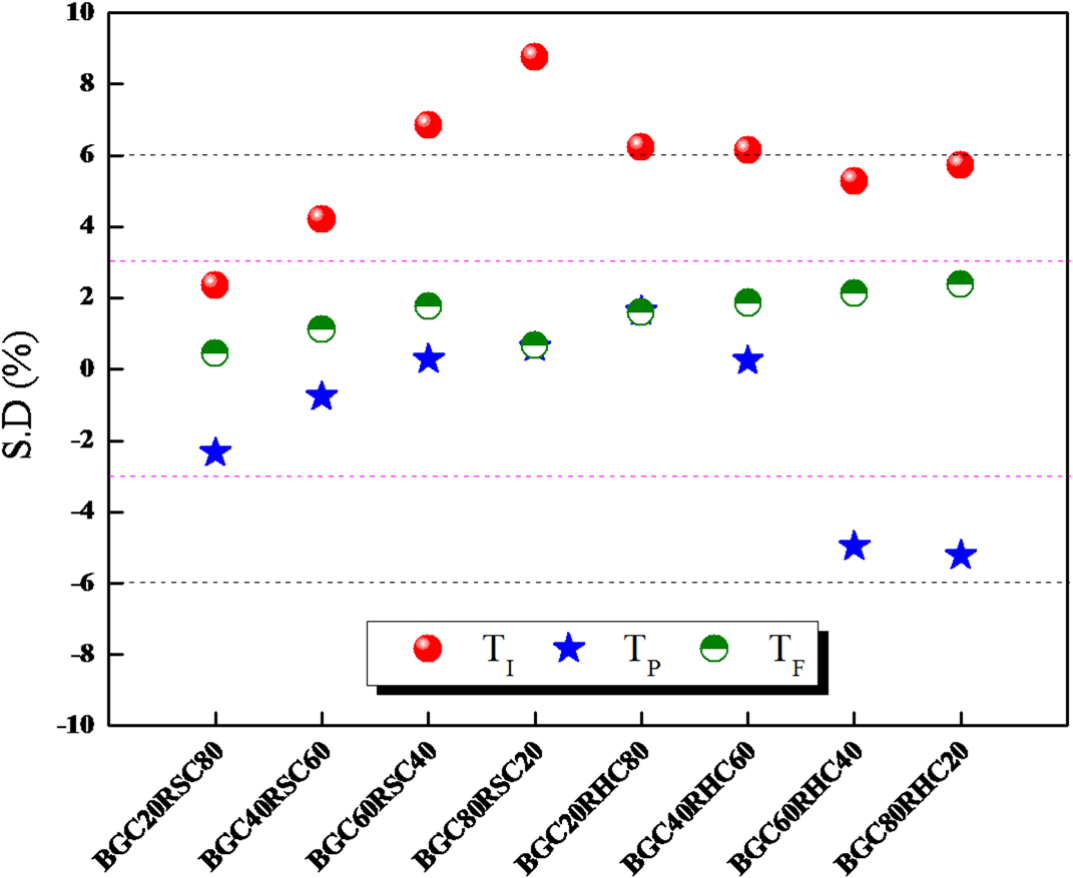
Standard deviation in experimental and theoretical combustion temperatures for different biochar blends.

In the above expressions, $\:{T}_{cal}$ is the calculated combustion temperature (T_I_, T_P_ or T_F_) for individual BGC or RSC or RHC, *w*_1_ is the mass fraction of RSC or RHC in the mixture, and *w*_*2*_ is the mass fraction of BGC in the mixture. Non-zero value of *S.D* (*S.D* > ± 3) infers possible interactions among the volatiles and char during the combustion of blends. Negative value of *S.D* infers beneficial synergistic effect, while positive *S.D* indicates non-beneficial interaction among fuels. Figure 5 shows the *S.D* values for different biochar blends. *S.D* of *T*_*I*_ is positive for all blends, which showcases the higher rate of release of lighter volatiles and subsequent heat transfer during combustion of blends. *S.D* of *T*_*P*_ is negative with high magnitude for BGC60RHC40 (−4.9%) and BGC80RHC20 (−5.2%), while the *S.D* of *T*_*F*_ is found to be well within 3% for all blends. Such a low and invariant *S.D* for *T*_*P*_ and *T*_*F*_ is due to the *T*_*P*_ - *T*_*F*_ thermal regime, which is mostly dominated by char combustion. This regime is also characterized by slower, bulk solid reactions and intrinsic reactivity of the feedstocks.

### Combustion kinetic study using DAEM method

Combustion kinetic modeling was performed using the DAEM approach with the assumption of two or three pseudocomponents ($\:{p}_{i}$) based on the nature of feedstock. These pseudocomponents include: (a) cellulose, hemicellulose and lignin for individual biomasses (BG, RS and RH), (b) partially decomposed lignin and carbonaceous matter (char) for biochars (BGC, RSC and RHC) and their blends (BGC: RSC blends and BGC: RHC blends), and (c) volatile matter and fixed carbon for coal. The pseudocomponents’ concentrations ($\:{p}_{i})$ were taken from the biochemical analysis (Table 1). Appropriate lower and upper bounds for the rate parameters (*E*_*a*_, *f* and σ) were taken from the literature^51–56^. For biomasses, biochars and biochar blends, the experimental values of conversion (*α*) and DTG were taken between 200 and 600 °C at 10 °C interval. Optimized rate parameters (*E*_*a*_, *f* and σ) for each pseudocomponent of biomasses, biochars and biochar blends are summarized in Table 4.

For RH combustion, *E*_*a*_ value was found to be higher for lignin (195.0 kJ mol^−1^) followed by cellulose (142.0 kJ mol^−1^), and minimum for hemicellulose (124.0 kJ mol^−1^) (Table 4). Such a variation in *E*_*a*_ among pseudocomponents is attributed to lignin having lower thermal reactivity and hemicellulose having higher thermal reactivity^22,57^. In addition, lignin is a branched and cross-linked phenolic polymer consisting of syringyl, guaiacyl and hydroxy phenyl monomer units linked by a variety of linkages like α-O-4, β-O-4, 4-O-5, 5–5, β-β, etc., with their bond dissociation energies varying in a wide range^58^. Irrespective of the feedstock, the value of *f* was assumed constant for each pseudocomponent. A similar approach was adopted by Ma et al. [^27^] and Chakraborty et al.^57^ for the combustion and pyrolysis kinetic study of biomasses. The value of *f* was highest for lignin (10^11.7^ min^−1^) and lowest for hemicellulose (10^9.1^ min^−1^).

The values of σ were slightly higher for lignin (6–10 kJ mol^−1^) compared to hemicellulose and cellulose, as lignin decomposition occurs over a broad temperature range (200–600 °C)^59^. For BG and RS, *E*_*a*_ values were similar for the pseudocomponents, which shows their similar influence on the biomass combustion. Furthermore, *E*_*a*_ value was lower for hemicellulose and lignin pseudocomponents of BG and RS compared to that for RH. The low values of *E*_*a*_ are due to the presence of higher hemicellulose and lower availability of lignin in BG and RS, as compared to RH. It is worthwhile to note that the sum of $\:{p}_{HC}$, $\:{p}_{CL}$ and $\:{p}_{L}$ is unity from Table 4, which means the relative amounts of hemicellulose, cellulose and lignin are scaled and presented on a dry ash free basis. The constant values of $\:{p}_{HC}$ for BG and RS, and $\:{p}_{CL}$ for BG and RH show that the hemicellulose and cellulose content in all three feedstocks are similar after correction for the ash content.

**Table 4:** Optimized kinetic parameters for combustion of individual biomasses, Biochars and Biochar blends.

Sample	$\:{p}_{HC}$ (wt/wt)	$\:{p}_{CL}$ (wt/wt)	$\:{p}_{L}$ (wt/wt)	$\:{p}_{CH}$ (wt/wt)	$\:{E}_{a,\:HC}$ (kJ mol^−1^)	$\:{E}_{a,\:CL}$ (kJ mol^−1^)	$\:{E}_{a,\:L}$ (kJ mol^−1^)	$\:{E}_{a,\:CH}$ (kJ mol^−1^)	$\:{\sigma\:}_{HC}$ (kJ mol^−1^)	$\:{\sigma\:}_{CL}$ (kJ mol^−1^)	$\:{\sigma\:}_{L}$ (kJ mol^−1^)	$\:{\sigma\:}_{CH}$ (kJ mol^−1^)
BG	0.29	0.48	0.23	-	110.0	142.0	180.9	-	5.0	4.0	6.0	-
RS	0.29	0.49	0.22		110.0	142.0	180.9		5.0	7.0	10.0	
RH	0.18	0.48	0.34		124.0	142.0	195.0		6.0	5.0	8.0	
BGC	-	-	0.74	0.26	-	-	175.5	247.6	-	-	17.0	8.0
RSC			0.19	0.81			130.8	247.6			20.0	8.0
RHC			0.35	0.65			158.9	247.6			17.0	10.0
BGC20RSC80	-	-	0.30	0.70	-	-	139.7	247.6	-	-	13.5	8.0
BGC40RSC60			0.41	0.59			148.7	247.6			13.5	8.0
BGC60RSC40			0.52	0.48			157.6	247.6			18.5	8.0
BGC80RSC20			0.63	0.37			166.6	247.6			18.5	8.0
BGC20RHC80	-	-	0.43	0.57	-	-	162.0	247.6	-	-	17.0	9.0
BGC40RHC60			0.51	0.49			165.5	247.6			17.0	9.0
BGC60RHC40			0.58	0.42			168.6	247.6			17.0	9.0
BGC80RHC20			0.66	0.34			172.0	247.6			17.0	9.0

*f_HC_- 10^9.1^ min^-1^, *f*_CL_- 10^10.4^ min^-1^, *f*_L_- 10^11.7^ min^-1^, *f*_CH_- 10^16.4^ min^-1^, HC - hemicellulose, CL - Cellulose, L - Lignin, CH - Char.

**Fig. 6:**
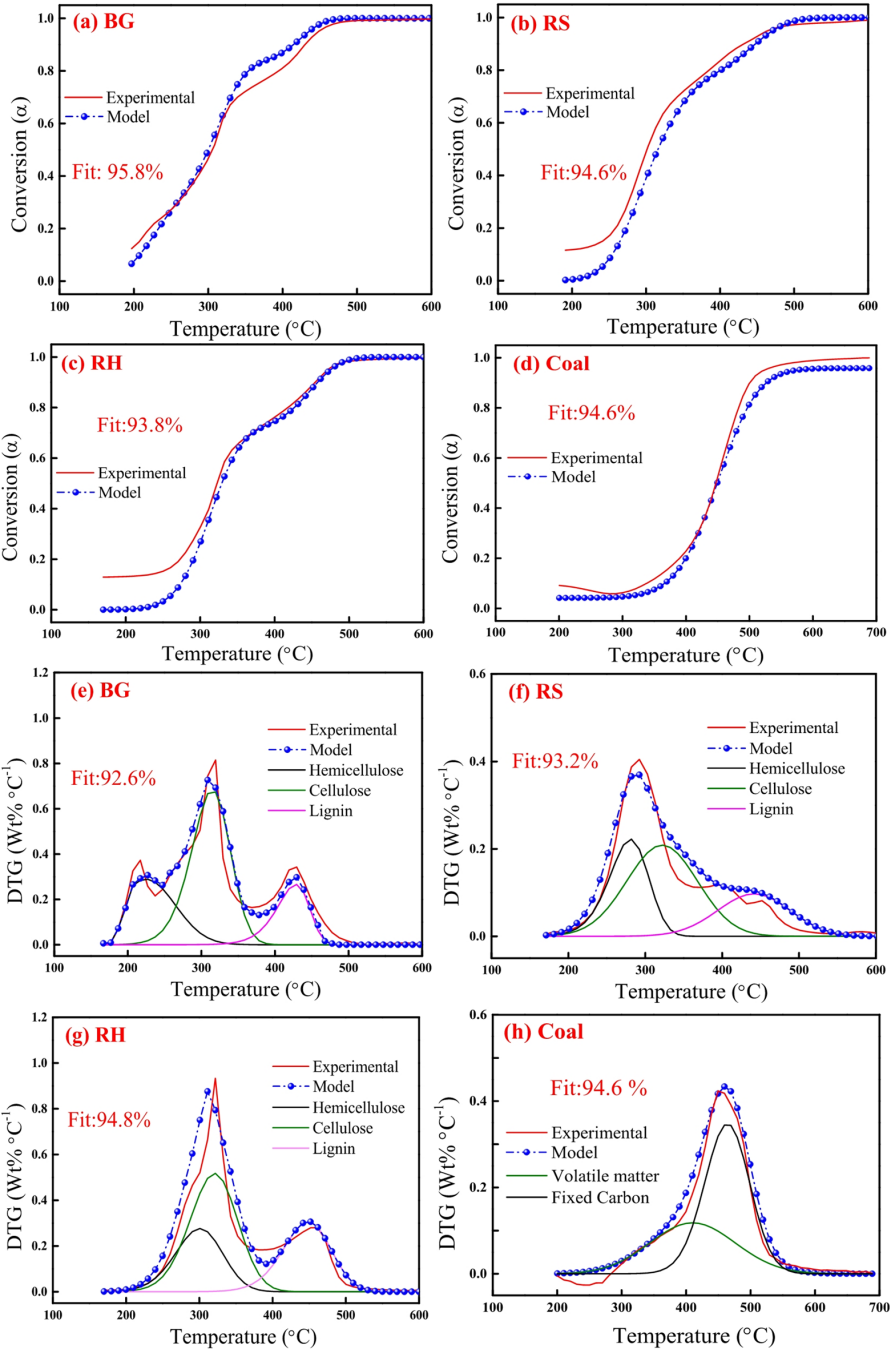
DAEM plots for mass conversion (*α*): **(a)** BG, **(b)** RS, **(c)** RH and **(d)** Coal, and *DTG*: **(e)** BG, **(f)** RS, **(g)** RH and **(h)** Coal at 10 °C min^−1^.

**Fig. 7:**
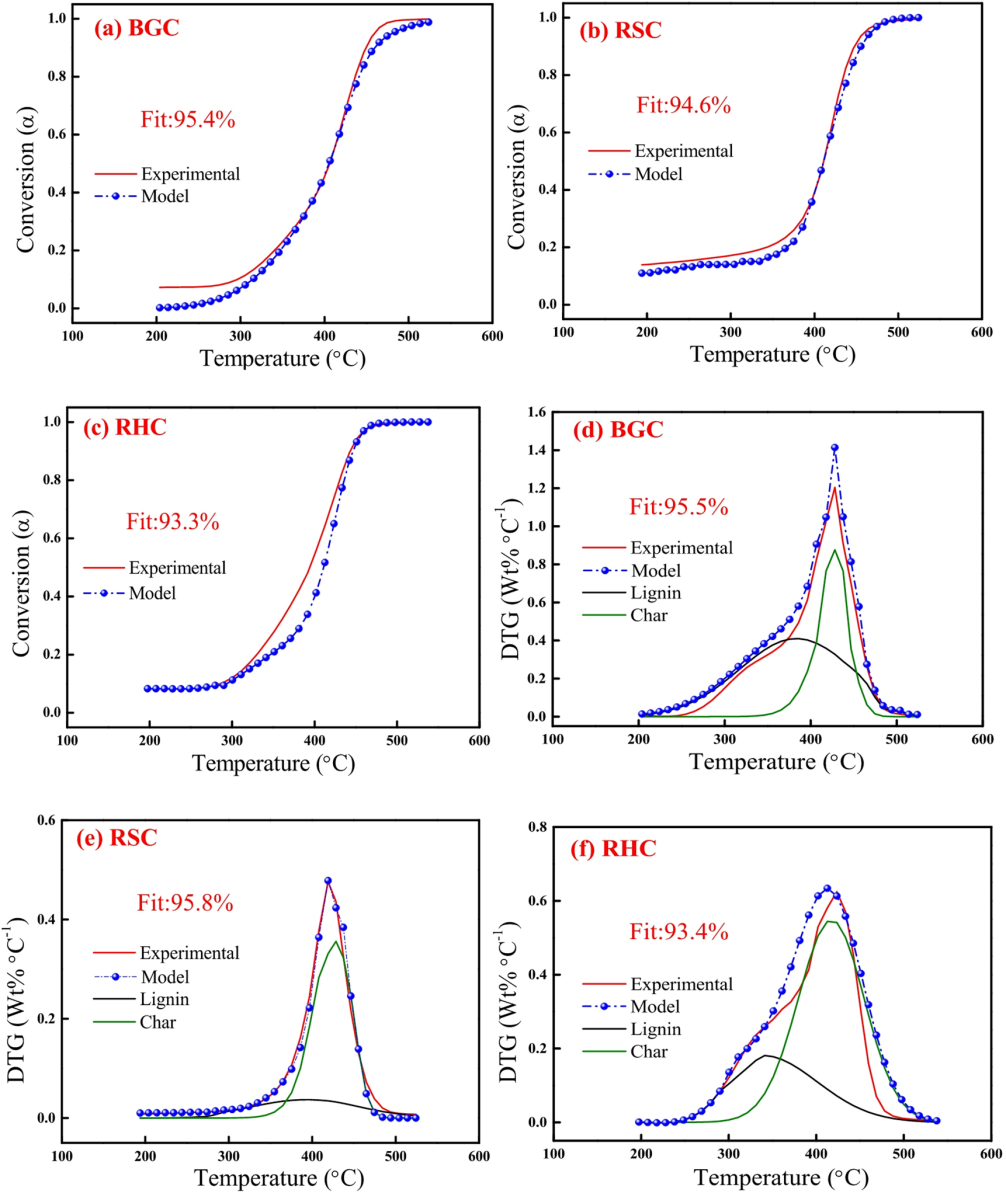
DAEM plots for mass conversion (*α*): **(a) **BGC, **(b)** RSC and **(c)** RHC, and *DTG*: **(d)** BGC, **(e)** RSC and **(f)** RHC at 10 °C min^−1^.

The pseudocomponents, viz., hemicellulose, cellulose and lignin, are useful to describe the decomposition of biomass by pyrolysis and combustion. However, biochar is depleted of cellulose and hemicellulose, and contains partially decomposed lignin and carbonaceous matter. Partially decomposed lignin corresponds to lignin-derived structures from primary pyrolysis reactions, especially before the occurrence of complete aromatization and condensation into stable char. Compared to raw lignin, partially decomposed lignin has higher aromaticity, reduced aliphatic side chains, and diminished oxygenated functionalities, and displays reduced solubility in conventional solvents due to increased molecular weight and crosslinking. Carbonaceous matter (Char) is the fully transformed fraction resulting from the aromatization, crosslinking and polycondensation in the solid residue. From the FTIR analysis, it is clear that the partially decomposed lignin mainly contains etheric C–O linkages along with aromatic C–H originating from the guaiacyl and syringyl units, while char contains C = C and C–H functionality associated with the aromatic units. These partially decomposed lignin ($\:{p}_{L})$ and carbonaceous matter or char ($\:{p}_{CH}$) compositions were estimated by correlating the sum of VM and FC present in a particular biomass and biochar. Here, the sum of $\:{p}_{L}$ and $\:{p}_{CH}$ in biochar is considered unity. Initially, the sum of VM and FC of biochar was multiplied with the biomass composition ($\:{p}_{HC}$, $\:{p}_{CL}\:\text{a}\text{n}\text{d}\:{p}_{L})$ to obtain the biochar composition ($\:{p}_{L}$and $\:{p}_{CH})$. $\:{p}_{L}$ concentration in biochar was determined by normalizing the lignin composition (i.e. by excluding hemicellulose and cellulose), because hemicellulose and cellulose are considered absent in biochar. On the other hand, $\:{p}_{CH}$ content was calculated by subtracting the $\:{p}_{L}$ content from 1, as the sum of $\:{p}_{L}$ and $\:{p}_{CH}$ is unity. A similar approach was used by Bach et al.^22^ to estimate the composition of torrefied biomass, where they considered char as one of the pseudocomponents. This extra pseudocomponent, called $\:{p}_{CH}$, along with $\:{p}_{L}$ is considered for the DAEM of biochar combustion. From Table 4, mass fractions of $\:{p}_{L}$ in biochars are noticeably high for BGC, RHC, while they are low for RSC. $\:{p}_{CH}$ value follows the trend: RSC (0.81) > RHC (0.65) > BGC (0.26).

For biochar combustion, *E*_*a*_ was found to be high for char and lowest for partially decomposed lignin. Such a variation in *E*_*a*_ is due to the thermal stability of char being comparatively higher than that of partially decomposed lignin. Furthermore, char exhibits stronger C = C stretching and aromatic C–H out-of-plane functional groups, with richer aromatic polycyclic structures and a higher degree of reticulation and thermal stability. This leads to higher bond dissociation energy of the molecules present in it ^60^. For all the three biochars, *E*_*a*_ was found to be similar (247.6 kJ mol^−1^) for char combustion signifying that alternation in $\:{p}_{CH}$ composition has insignificant impact on the biochar thermal stability. The major variation in *E*_*a*_ was observed for the combustion of partially decomposed lignin present in different biochars. For this pseudocomponent, *E*_*a*_ was maximum for BGC (175.5 kJ mol^−1^) and minimum for RSC (130.8 kJ mol^−1^). Interestingly, this parameter follows a linear trend in biochars, which suggests that a higher fraction of partially decomposed lignin in biochar results in a higher E_a_. In this regard, the kinetic parameter obtained from the DAEM method is valuable because it presents a range of activation energies that are well correlated to each pseudocomponent. For biochar combustion, the parameter *f* was highest for char (10^16.4^ min^−1^) and lowest for partially decomposed lignin (10^11.7^ min^−1^). For biochars, σ was found to be higher for the partially decomposed lignin pseudocomponent (17–20 kJ mol^−1^) and minimum for char pseudocomponent (8.0–10.0 kJ mol^−1^), because even partially decomposed lignin has a more complex structure with widely varying bond dissociation energies, and a certain degree of aromatization.

**Fig. 8:**
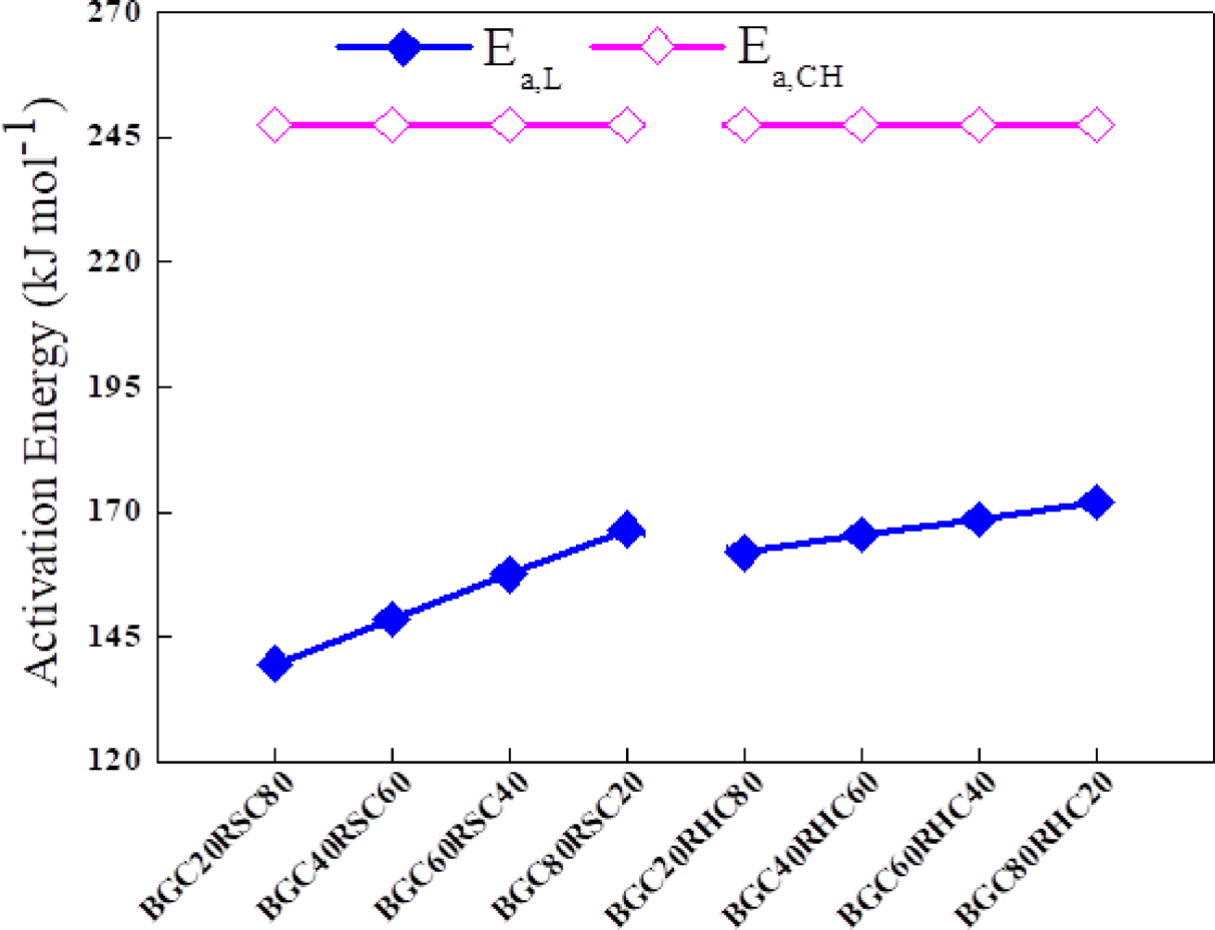
Variation of pseudocomponents’ activation energy with blend composition.

Figures 6 and 7 depict the experimental and model profiles of α and DTG with temperature for biomasses (BG, RS and RH), biochars (BGC, RSC and RHC) and coal based on the optimized kinetic parameters at 10 °C min^−1^. It is evident that the model DTG plots match well with experimental DTG and the pseudocomponent profiles are reasonably well captured. Notably, the fit% was found to be reasonably good (93–96%) for both α and DTG plots of BG, RS, RH, BGC, RSC and RHC. The validity of the optimized kinetic parameters at 10 °C min^−1^ was also examined for the α and DTG profiles at 20 °C min^−1^, which is given in Figures S2, S3 (in Supplementary Material), where the fit% values are high (91–98%).

For BGC: RSC and BGC: RHC blends, the $\:{\text{E}}_{\text{a},\text{C}\text{H}}$ value corresponding to char pseudocomponent is unaltered with BGC weight fraction in blend. This may be attributed to minimal variation in C = C and aromatic C–H functional groups with the variation of BGC mass fraction in blend, as qualitatively evidenced from the FTIR spectroscopic analysis. Such findings signify non-beneficial interactions between the intermediates produced from aromatic polycyclic structures of the char. More importantly, the variations in the DTG and conversion profiles of the blends are well captured by the variation of $\:{\text{E}}_{\text{a},\text{L}}$ corresponding to the combustion of partially decomposed lignin. For both BGC: RSC and BGC: RHC blends, $\:{\text{E}}_{\text{a},\text{L}}$ increased as BGC content increased in the blend. This can be correlated, atleast partially, to the increase in peak intensity of C–O stretching and aromatic C–H in-plane bending vibrations. As shown in Table 4, $\:{p}_{L}$ and $\:{p}_{CH}$ values vary as per the blend mass fraction, and their sum is unity. The $\:{\text{E}}_{\text{a}}$ variations are also well within the bounds of activation energies of lignin in these three individual biochars. Figure 8 depicts the variation of activation energy with the change of mass fraction of individual biochars in the blend. The linear increase of $\:{\text{E}}_{\text{a},\text{L}}$ in BGC: RSC and BGC: RHC with respect to RSC and RHC contents is an interesting observation. This only points to the increase in the fraction of partially decomposed lignin as the BGC content increases in the blend. As compared to RSC, the presence of RHC enhances the magnitude of $\:{\text{E}}_{\text{a},\text{L}}$, which shows the critical role of extent of carbonization on the activation energy of combustion of the biochars. This also correlates well with Table 1, where RHC exhibits a better carbon and FC content than RSC.

**Fig. 9:**
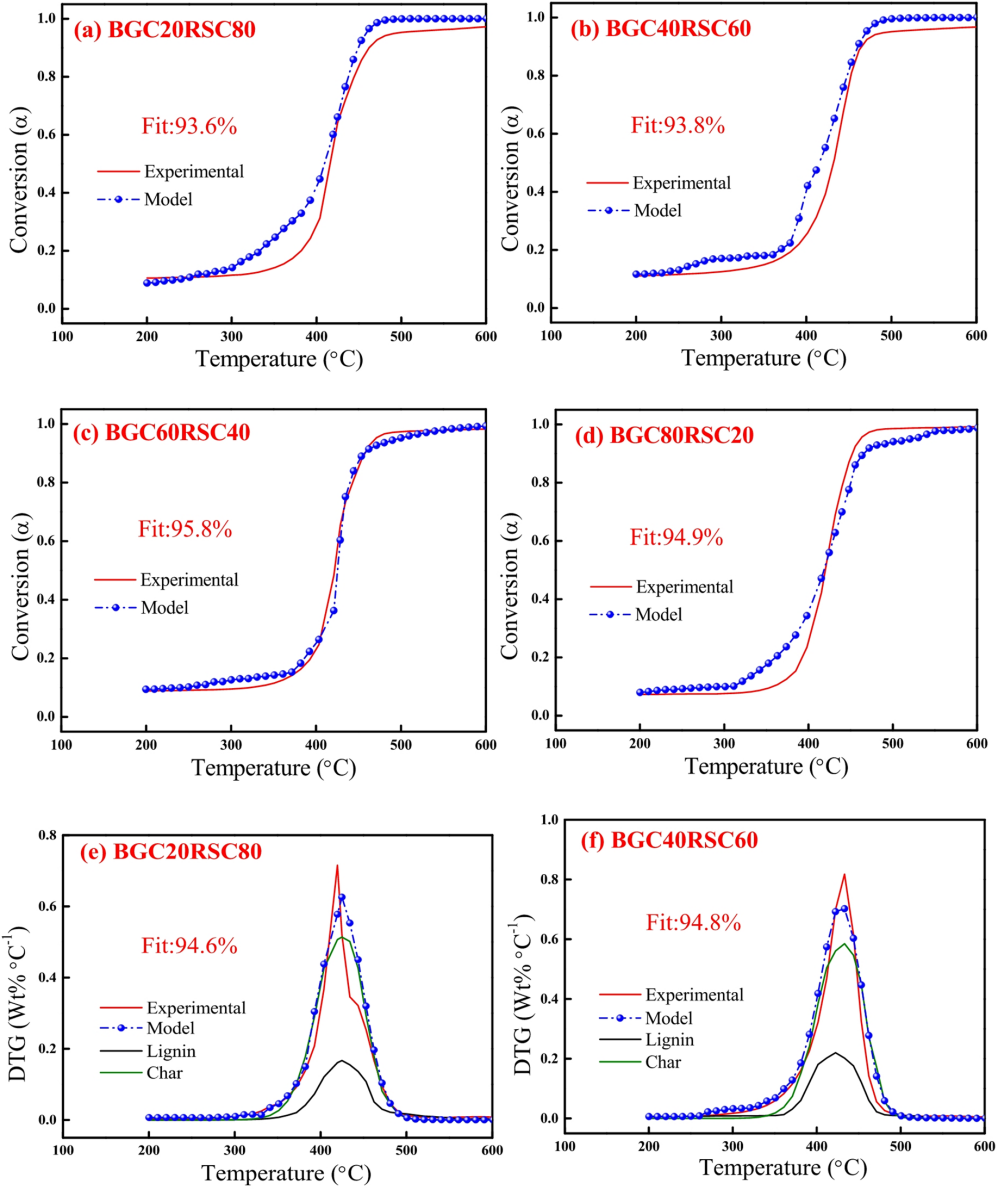

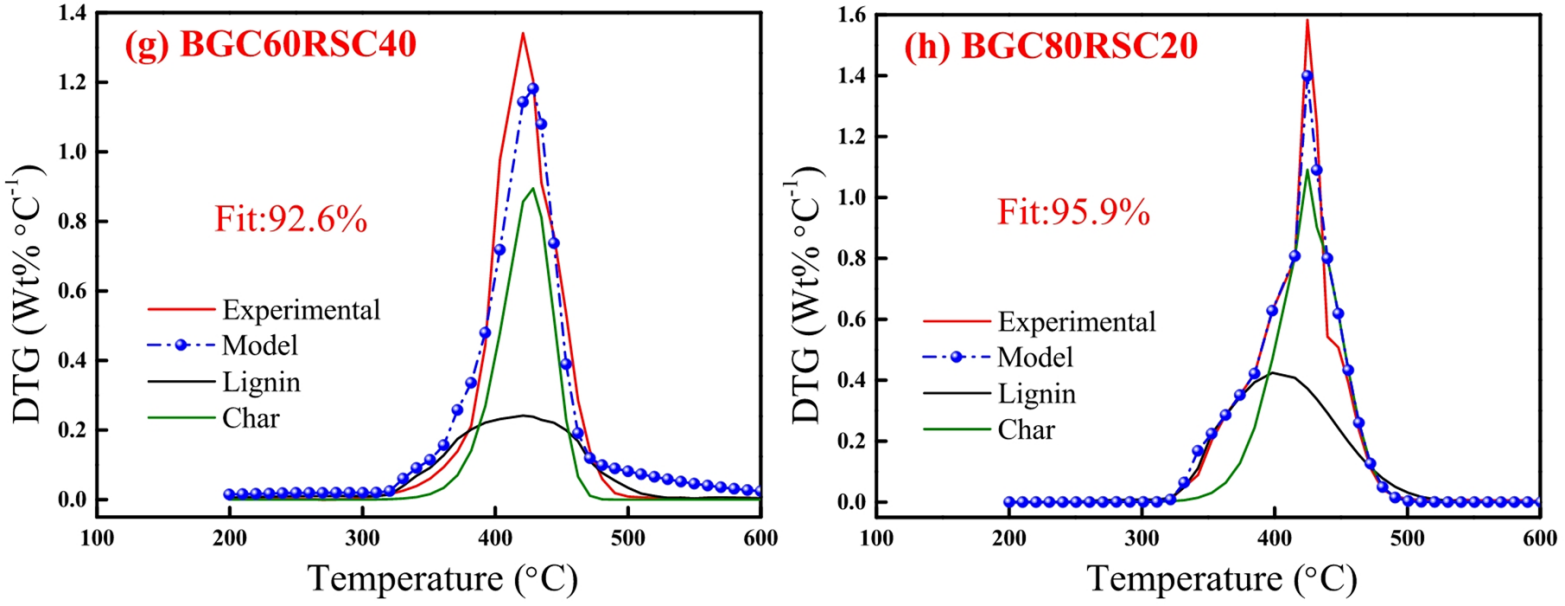
DAEM plots for mass conversion (*α*): **(a)** BGC20RSC80, **(b)** BGC40RSC60, **(c)** BGC60RSC40 and **(d)** BGC80RSC20, and *DTG*: **(e)** BGC20RSC80, **(f)** BGC40RSC60, **(g)** BGC60RSC40 and **(h)** BGC80RSC20 at 10 °C min^−1^.

**Fig. 10:**
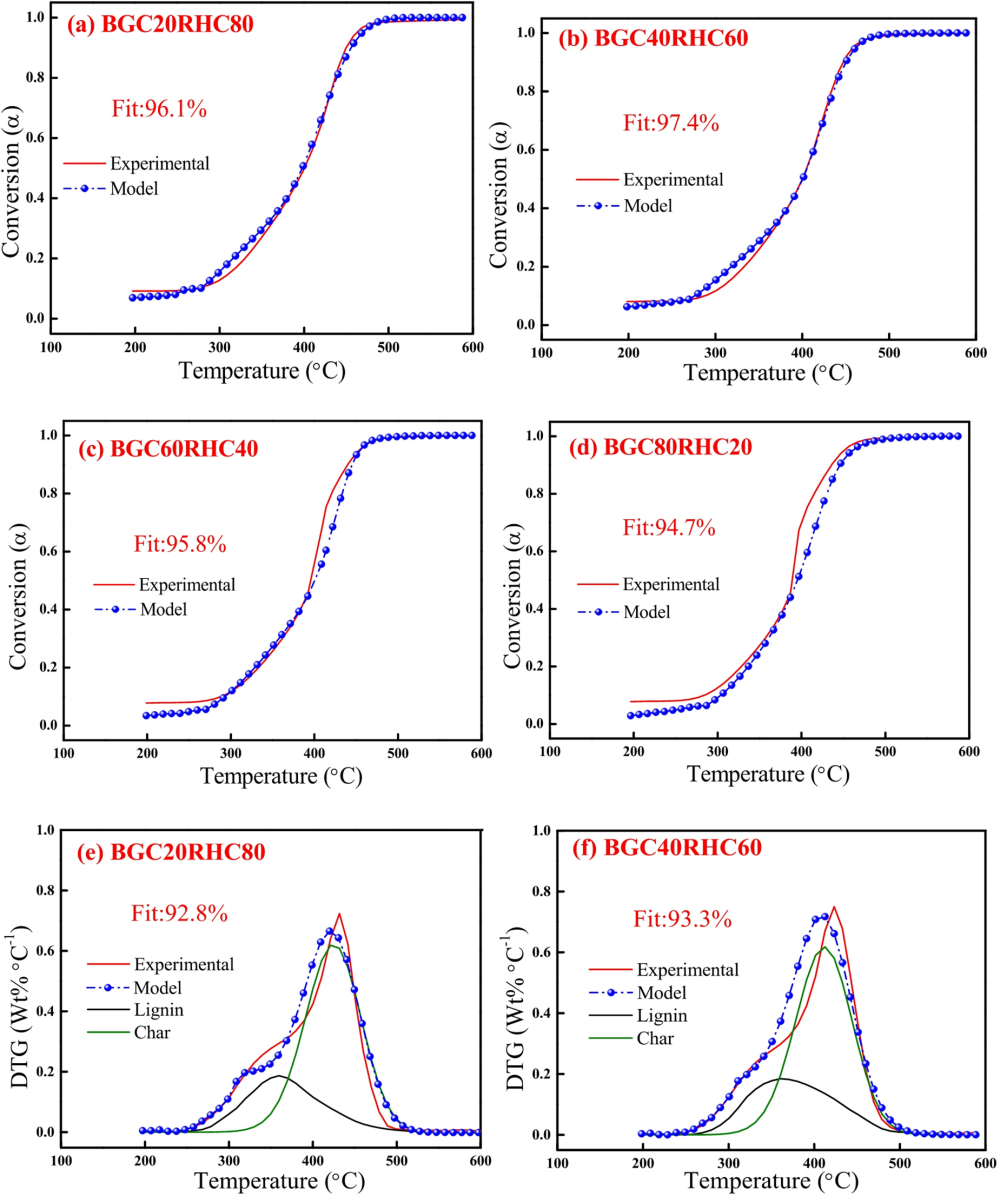

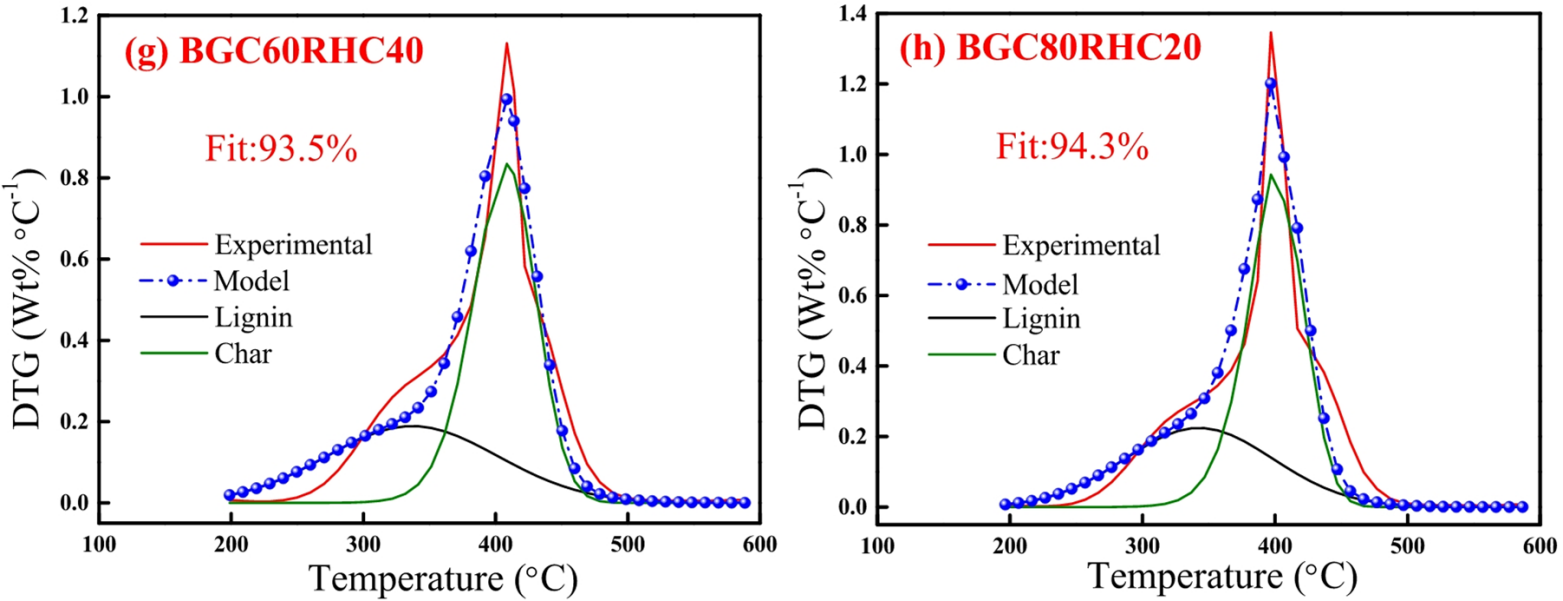
DAEM plots for mass conversion (*α*): **(a)** BGC20RHC80, **(b)** BGC40RHC60, **(c) **BGC60RHC40 and **(d)** BGC80RHC20, and *DTG*: **(e)** BGC20RHC80, **(f)** BGC40RHC60, **(g)** BGC60RHC40 and **(h)** BGC80RHC20 at 10 °C min^−1^.

**Fig. 11:**
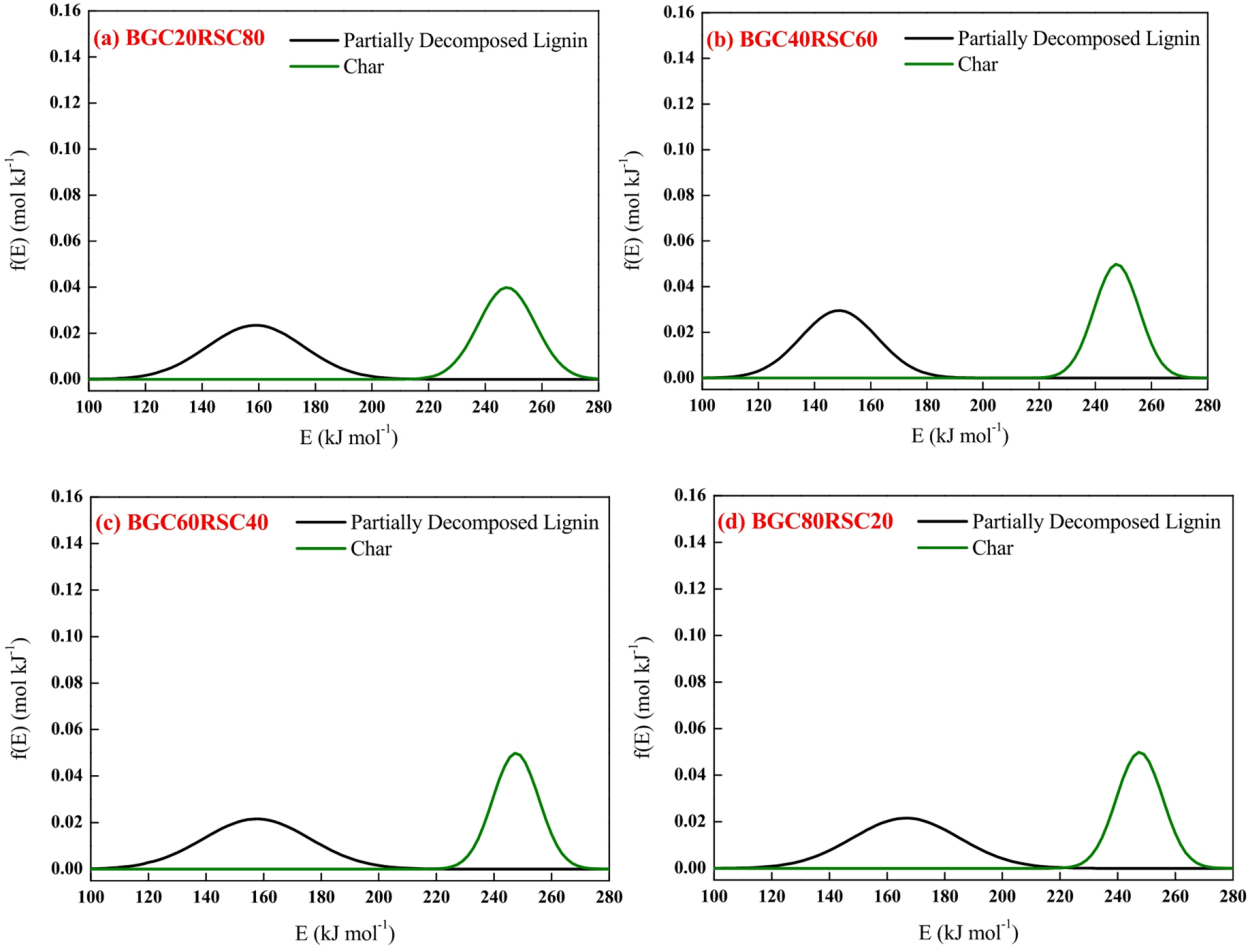

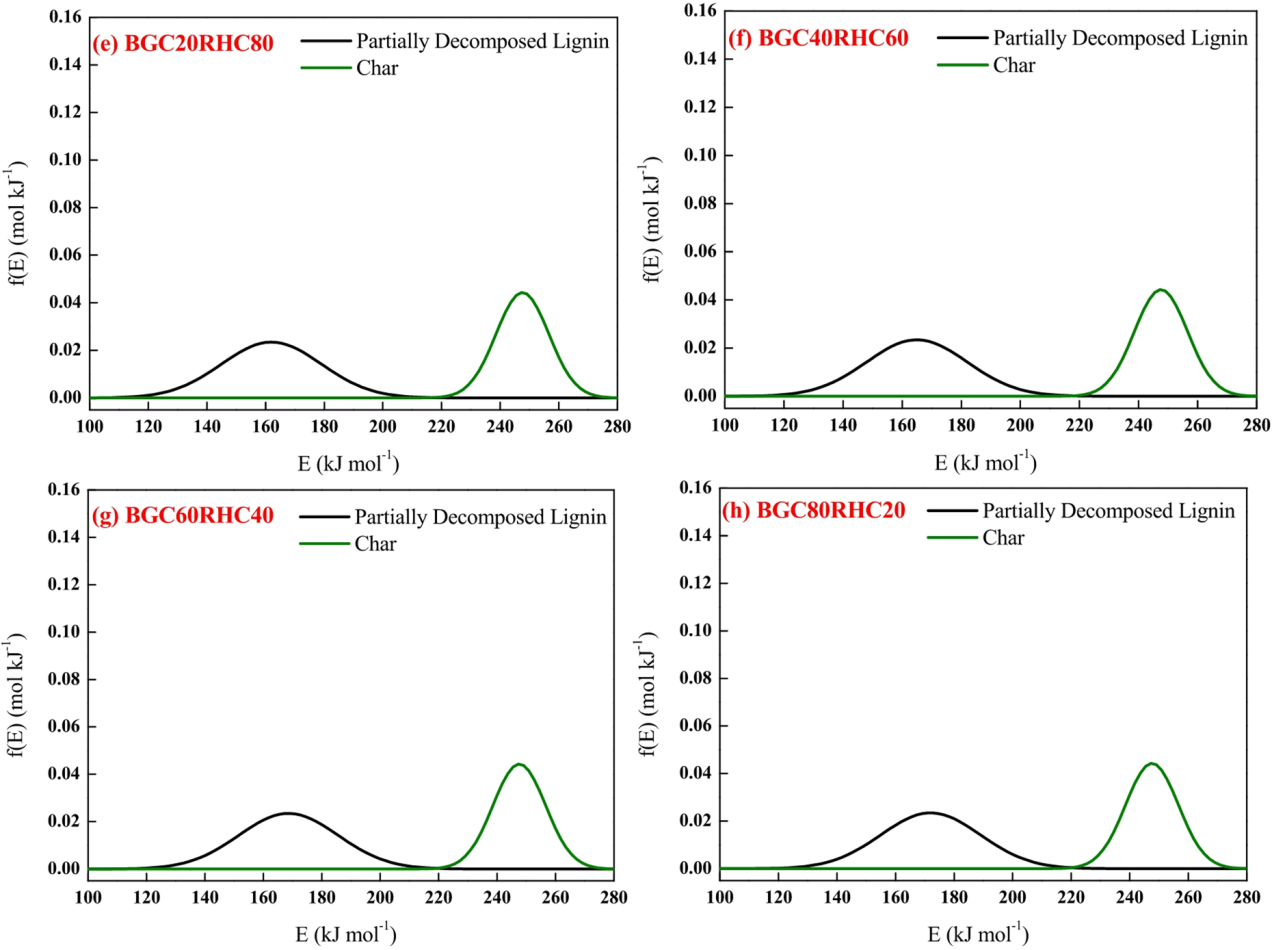
Activation energy distribution for different pseudocomponents in the decomposition of **(a)** BGC20RSC80, **(b)** BGC40RSC60, **(c)** BGC60RSC40, **(d)** BGC80RSC20, **(e)** BGC20RHC80, **(f)** BGC40RHC60, **(g)** BGC60RHC40 and **(h)** BGC80RHC20.

Figures 9 and 10 depict the experimental and model profiles for α and DTG with temperature for BGC: RSC and BGC: RHC blends at 10 °C min^−1^. It is evident that the fit% is good for conversion (~ 93–97%) and DTG profiles (~ 92–96%). Such a good fit, generated by altering the activation energy in accordance with the simple binary mixture rule, signifies the robustness of DAEM, and its possible adoption for combustion of different lignocellulosic biochar blends. While there are no previous reports on the kinetic parameters of biochar combustion, especially of the biochar blends using a robust DAEM, Liu et al.^61^ estimated the combustion kinetics of blended solid fuels using the Arrhenius equation and reported that blend’s activation energy follows the mixture rule. Vhathvarothai et al.^10^ reported the applicability of a similar mixture rule for the decomposition of coal-biomass blends using Kissinger method. The validity of the DAEM parameters was also examined for the α and DTG of blends at 20 °C min^−1^, as illustrated in Figures S4, S5 (in Supplementary Material). The model fit% values are good (91–96%), which demonstrates the robustness of the rate parameters even at a different heating rate. Figure 11 depicts the Gaussian distribution of activation energies associated partially decomposed lignin and char for the combustion of blends of BGC-RSC and BGC-RHC.

It is important to note that with the pseudocomponents, viz., partially decomposed lignin and char, DTG and conversion profiles are reasonably well captured by the model for biochar blends. It is worthwhile to note that the catalytic role of ash on the decomposition rate and conversion of the biochar blends is not incorporated in the model. The ash may affect the reactivity of the blends especially at high combustion temperatures (> 1000 °C), as temperatures above the ash flow point promote the development of different eutectic materials and complexes in the molten ash, along with elevated carbon conversion. At lower temperatures, the ash has minimal impact on the combustion reactivity ^62^. It should be noted that the model used in this study is solely based on the intrinsic kinetics of biochar blends during combustion without accounting the heat and mass transfer effects, which may influence the process at larger scales. Thus, the chemical kinetic regime is characteristic of small particle sizes, typical of fluidized bed and entrained flow combustors, and lower temperature regimes corresponding to carbon conversion in the raw material. Overall, the *E*_*a*_ of RSC, RHC and blends of 20–40 wt% BGC with 60–80 wt% RSC or RHC are somewhat similar to that of coal (145.3 kJ mol^−1^ for VM and 260.9 kJ mol^−1^ for FC pseudocomponent). Therefore, RSC, RHC and the binary blends of BGC: RSC and BGC: RHC can be considered as possible substitute for coal in the thermal utilities of industries without requiring significant modifications to the main reactor.

### Burning time, heat release, fuel consumption and Ash generation

Feedstocks getting processed in the thermal utility must possess an adequate heat release rate in order to maintain the reactor temperature at the optimum level. From this point of view, heat release rate ($\:{H}_{RT})$ and minimum burning time ($\:{M}_{BT}$) for a unit amount of feedstock were estimated based on $\:{DTG}_{max}$ and HHV using Eqs. (14–15). In a reactor, all fuel particles experience similar time duration to get effectively combusted. The burning duration depends on turbulence and the settling velocity of fuel particles inside the reactor. All fuel particles must be burnt before exiting the reactor in the form of ash. With the change of physical and chemical characteristics of the feedstock, the particles may not get sufficient time to burn properly. Thus, $\:{M}_{BT}$ is a critical parameter to estimate the burning efficiency of a fuel. Theoretically, $\:{M}_{BT}$ is the shortest duration required for a fuel particle to undergo complete combustion. Similarly, $\:{H}_{RT}$ is the associated heat release rate during combustion^63^.

14 $$\:{\text{M}}_{\text{B}\text{T}}\left(\text{m}\text{i}\text{n}\right)=\frac{100}{{\text{D}\text{T}\text{G}}_{\text{m}\text{a}\text{x}}\times\:10}$$

15 $$\:{\text{H}}_{\text{R}\text{T}}\left(\text{M}\text{J}\:{\text{m}\text{i}\text{n}}^{-1}\right)=\:\frac{{\text{D}\text{T}\text{G}}_{\text{m}\text{a}\text{x}}\times\:10\times\:\text{H}\text{H}\text{V}}{100}$$

**Fig. 12:**
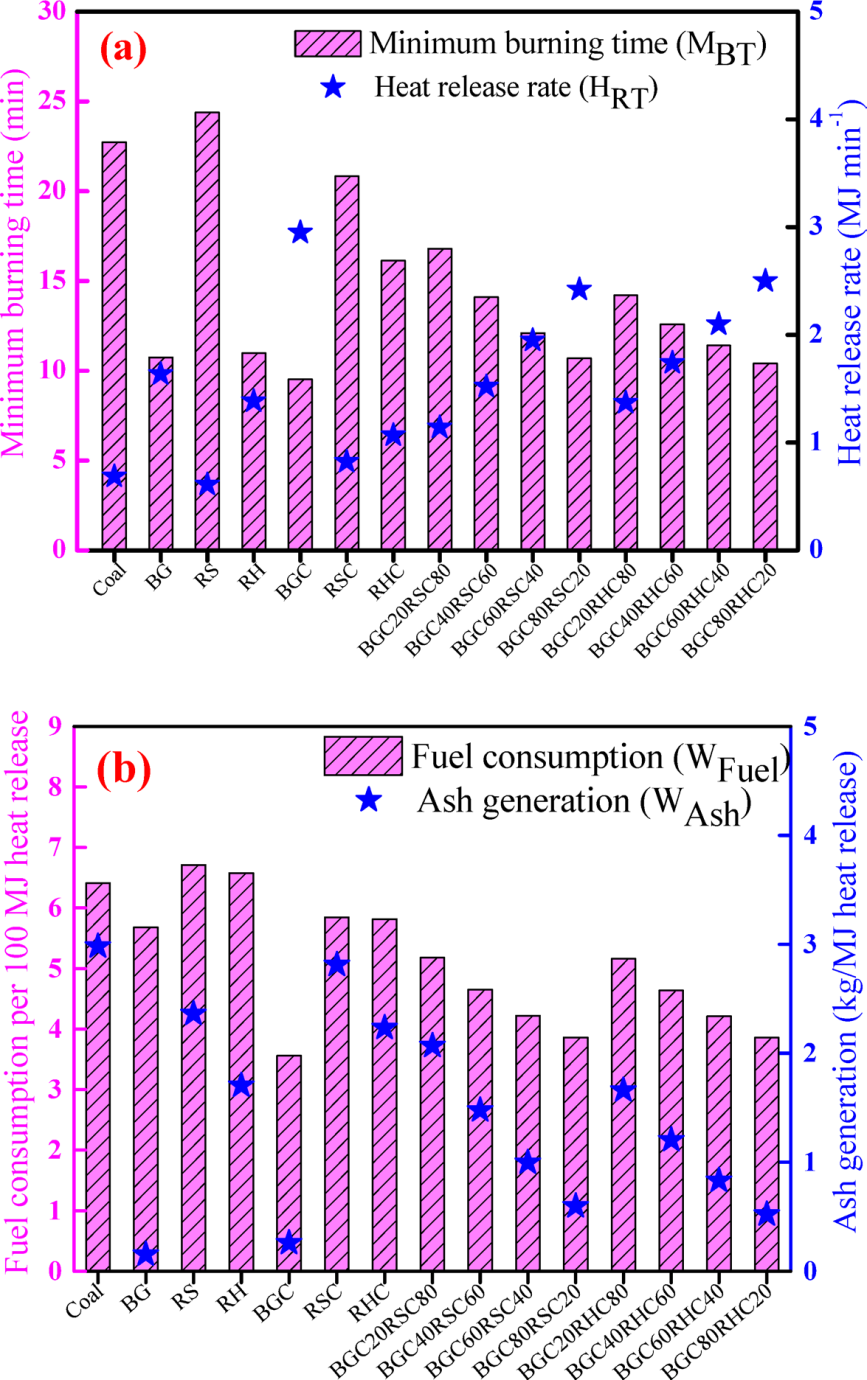
Variation of **(a)** minimum burning time and heat release rate, and **(b)** fuel consumption and ash generation for coal, biomass, biochar and blends.

Figure 12a illustrates the variation of $\:{H}_{RT}$ and $\:{M}_{BT}$ for different feedstocks. $\:{M}_{BT}$ is minimum for BGC (9.5 min) and highest for RS (24.4 min). $\:{M}_{BT}$ for RSC (20.8 min) is quite closer to coal (22.7 min). For BGC: RSC blend, with the increase of BGC mass fraction from 20 wt% (BGC20RSC80) to 80 wt% (BGC80RSC20), $\:{M}_{BT}$ decreases from 18.6 min to 11.8 min. A similar observation is evident for BGC: RHC blend. Such a fall in $\:{M}_{BT}$ with the rise in BGC fraction is attributed to decline in ash content and increase in volatile matter, which promotes the ignition characteristics. $\:{H}_{RT}$ is high for BGC, while it is low for coal. $\:{H}_{RT}$ for RSC is similar to coal. For BGC: RHC blend, $\:{H}_{RT}$ increases from 1.4 MJ min^−1^ to 2.6 MJ min^−1^ with the increase of BGC mass fraction from 20 wt% to 80 wt%. A similar variation in $\:{H}_{RT}$ is observed for BGC: RSC blend.

The increase in $\:{H}_{RT}$ is attributed to the decline in ash content and increase in combustible matter, and higher HHV of BGC. Due to variations in ash, HHV and $\:{H}_{RT}$, the required fuel consumption and the associated ash generation rate fluctuate in order to provide the constant energy input to the reactor. Fuel consumption ($\:{W}_{Fuel}$) and ash generation ($\:{W}_{Ash})$were estimated for the generation of 100 MJ heat from the feedstocks using the following Eqs. ^20^.

16 $$\:{\text{W}}_{\text{F}\text{u}\text{e}\text{l}}\left(kg\right)=\:\frac{100}{\text{H}\text{H}\text{V}}\:$$

17 $$\:{\text{W}}_{\text{A}\text{s}\text{h}}\left(kg\right)=\:\frac{\text{A}\text{s}\text{h}\:\left(\%\right)\times\:{\text{W}}_{\text{F}\text{u}\text{e}\text{l}}}{\text{H}\text{H}\text{V}}\:$$

Figure 12b depicts the variation of $\:{W}_{Fuel}$ and $\:{W}_{Ash}$ for different feedstocks. $\:{W}_{Fuel}$ for RS (6.7 kg), RH (6.6 kg) and coal (6.4 kg) are similar. Kumar and Nandi^20^ investigated the wheat straw and coal combustion behaviour, and estimated the fuel consumption rate for boilers. The consumption rate of biochar blends was quite similar to wheat straw-coal consumption rate reported by them. After pyrolysis, $\:{W}_{Fuel}$ is significantly reduced for each biochar. $\:{W}_{Fuel}$ for BGC is minimum compared to other biochars. For BGC: RSC blends, with the increase of BGC mass fraction from 20 wt% (BGC20RSC80) to 80 wt% (BGC80RSC20), $\:{W}_{Fuel}$ significantly decreases from 5.4 kg to 4.0 kg. A similar observation is valid for BGC: RHC blend. Ash generation ($\:{W}_{Ash}$) is minimum for BGC and maximum for coal, whereas, $\:{W}_{Ash}$ for RSC is quite similar to coal. For blends, $\:{W}_{Ash}$ decreases significantly with increasing BGC mass fraction. In a combustor, fuel particles should burn completely before they reach the outlet , so that they do not end up as unburnt ash. Different feedstocks react with oxygen in different ways based on the type of combustible and non-combustible materials they contain, and as a result, heat release rate and burning time differ depending on the feedstock characteristics. During the combustion of feedstocks containing different ash and HHV , fuel consumption and ash generation rates vary significantly. Hence, physico-chemical properties such as ash content, HHV, and TG-based combustion characteristics such as *DTG*_*max*_ play critical role . Therefore, in order to ensure efficient feedstock consumption in thermal utilities, this study emphasizes the need to analyze the parameters such as burning time, heat release, fuel consumption and ash generation rates. Overall, it can be concluded that individual RSC, RHC, and 20–40 wt% BGC blends with RSC or RHC exhibit characteristics potentially compatible with existing thermal utilities. However, these findings should be validated under pilot or full-scale conditions to account for particle-scale, flow-field, and heat/mass transfer effects.

### Limitations and future perspectives

The DAEM presented in this study focused solely on intrinsic kinetics by excluding heat and mass transport effects that is supposed to be relevant at a larger scale. Importantly, with two pseudocomponents, the DTG and conversion profiles for biochar blends were captured reasonably, even without accounting for ash and its catalytic role, which is only expected to be minimal at low temperatures but significant above 1000 °C. Even though this study is limited to three biochars (sugarcane bagasse, rice straw, and rice husk) and their binary blends, the results are indeed applicable when the feed particle sizes are small as in fluidized bed or entrained flow combustors, especially at temperatures below 700 °C. The future works must possibly include a wider range of heating rates (5–100 °C min^−1^), and bigger particle size of the feedstocks (few mm – 1 cm) to investigate the kinetics using DAEM approach, while also integrating the heat and mass transfer effects. It is also imperative to evaluate a wide range of feedstocks, including biochars derived from different biomass feedstocks of varying characteristics or ternary blends. The role of key ash constituents in altering the combustion behaviour also needs a thorough understanding by using model constituents such as lignin-derived oligomers and aromatized species.

## Conclusion

This study investigated the combustion characteristics and kinetics of biochars and their binary blends, comparing them with biomasses and coal. The TG-DTG profiles and activation energy values signify that the combustion of biochars and biochar blends is more stable and mature compared to biomass, and is similar to that of coal. The distributed activation energy model was employed to estimate the combustion kinetics of coal, biomass, biochar and biochar binary mixtures. The DAEM incorporating two pseudocomponents, viz., partially decomposed lignin and char, is shown to precisely capture the combustion kinetics of biochars and their blends. The model’s robustness was determined by applying the optimized parameters obtained from the training dataset at 10 °C min^−1^ to the data at 20 °C min^−1^, which was used for prediction. The model demonstrated good predictive accuracy, with a fit percentage exceeding 91%. This study highlights a methodology for arriving at the best set of kinetic parameters of combustion by focusing only on the sensitive pseudocomponents, such as partially decomposed lignin, to precisely elucidate the combustion kinetics of individual biochars and biochar blends. For biochars and biochar blends, the activation energy was minimum for partially decomposed lignin and maximum for char pseudocomponent (247.6 kJ mol^−1^). Notably, the activation energy of combustion for the char pseudocomponent remained constant, while that for partially degraded lignin pseudocomponent varied with the BGC fraction in the blend. The activation energy of combustion of biochar blends followed a weighted average rule without exhibiting synergistic interactions, indicating that the kinetic parameters of any lignocellulosic biochar mixtures can be predicted using the kinetic parameters of individual biochars. Based on the DAEM kinetic parameter estimation methodology reported in this study, it can be concluded that, for any biochar blends, assessing the kinetics of the individual biochars is sufficient to estimate the combustion behaviour of the blends. Overall, the DAEM and the proposed methodology offer a flexible framework for biochar combustion analysis, although careful calibration and validation with experimental data for specific biochars remain essential. The minimum burning time (20.8 min), heat release rate (0.82 MJ min^−1^), fuel consumption (5.85 kg) and ash generation rate (2.81 kg) of RSC are quite similar to coal. For BGC: RSC blends and BGC: RHC blends, fuel consumption, ash generation rate and burning time decreased significantly as BGC fraction increased in the blends. Overall, the findings demonstrate that biochars and their blends have the potential to serve as coal substitutes in existing thermal utilities, though practical implementation would require further validation under industrial operating conditions.

## Supplementary Information

Below is the link to the electronic supplementary material.

Supplementary Material 1

## Data Availability

The datasets used and/or analysed during the current study available from the corresponding author on reasonable request.
