# Supplementary material for: Distributed activation energy kinetic modeling of combustion of bagasse char, rice straw char and rice husk char blends
Source: Sci Rep. 2025 Nov 26;15:42033. doi: 10.1038/s41598-025-24976-8 (PMC12658152; doi:10.1038/s41598-025-24976-8)
Supplement: Supplementary file 1 — Supplementary Material 1 [file 41598_2025_24976_MOESM1_ESM.docx]

**Distributed Activation Energy Kinetic Modeling of Combustion of Bagasse Char, Rice Straw Char and Rice Husk Char Blends**

Pritam Kumar^a,b^*, Piyush Chaunsali^b^ and Ravikrishnan Vinu^a^*

^a^ Chemical Engineering Department, Indian Institute of Technology Madras, India, 600036

^b^ Civil Engineering Department, Indian Institute of Technology Madras, India, 600036


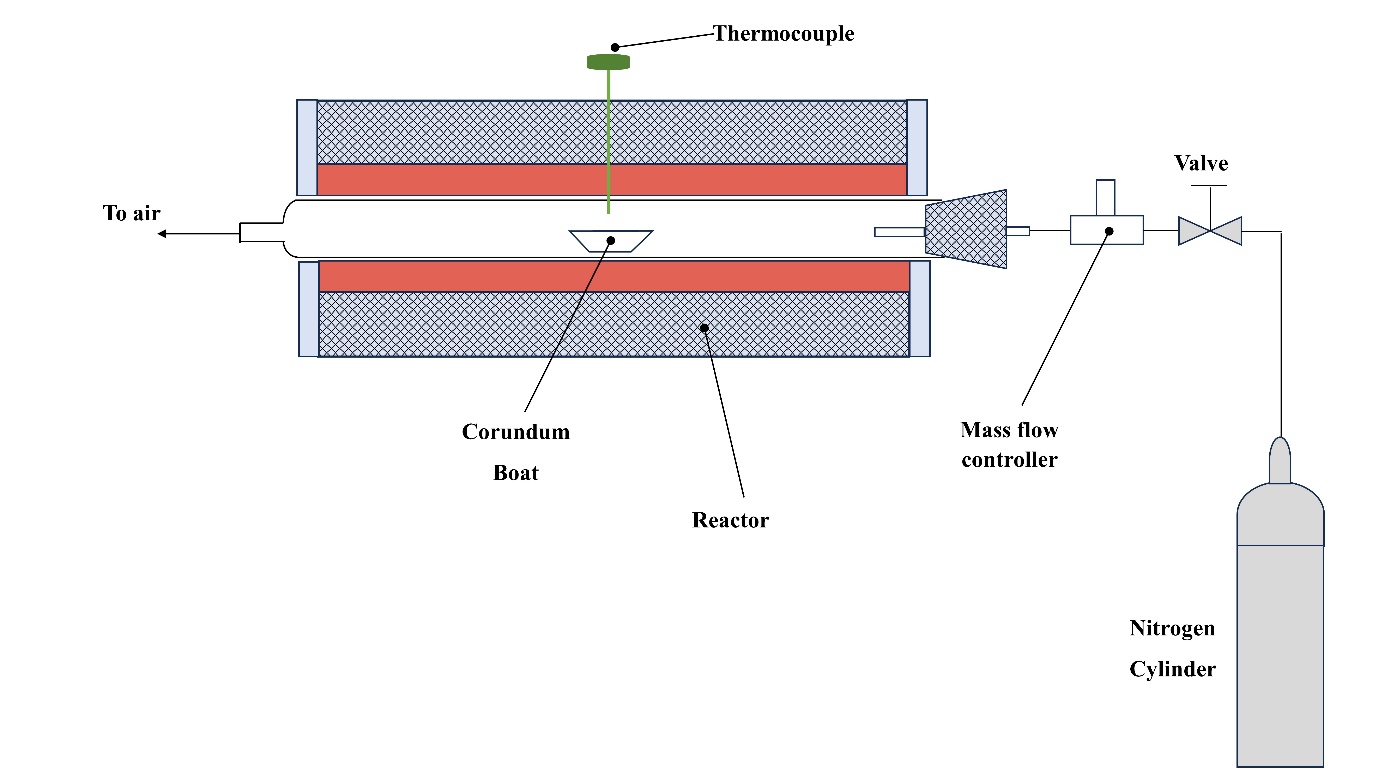


**Figure S1**: Schematic of the reactor setup for biochar production.

**Figure S2:** DAEM plots for mass conversion (α): (a) BG, (b) RS, (c) RH and (d) Coal, and DTG: (e) BG, (f) RS, (g) RH and (h) Coal at 20°C min^-1^.

**Figure S3:** DAEM plots for mass conversion (α): (a) BGC, (b) RSC and (c) RHC, and DTG: (d) BGC, (e) RSC and (f) RHC at 20°C min^-1^.

**Figure S4:** DAEM plots for mass conversion (α): (a) BGC20RSC80, (b) BGC40RSC60, (c) BGC60RSC40 and (d) BGC80RSC20, and DTG: (e) BGC20RSC80, (f) BGC40RSC60, (g) BGC60RSC40 and (h) BGC80RSC20 at 20°C min^-1^.

**Figure S5:** DAEM plots for mass conversion (α): (a) BGC20RHC80, (b) BGC40RHC60, (c) BGC60RHC40 and (d) BGC80RHC20, and DTG: (e) BGC20RHC80, (f) BGC40RHC60, (g) BGC60RHC40 and (h) BGC80RHC20 at 20°C min^-1^.
